# Disordered hinge regions of the AP-3 adaptor complex promote vesicle budding from the late Golgi in yeast

**DOI:** 10.1242/jcs.262234

**Published:** 2024-11-08

**Authors:** Mitchell Leih, Rachael L. Plemel, Matt West, Cortney G. Angers, Alexey J. Merz, Greg Odorizzi

**Affiliations:** ^1^ Department of Molecular, Cellular, and Developmental Biology, University of Colorado, Boulder, CO 80309, USA; ^2^ Department of Biochemistry, University of Washington, Seattle, WA 98195, USA

**Keywords:** Adaptor, Vesicle, Golgi, Membrane, Transport

## Abstract

Vesicles bud from maturing Golgi cisternae in a programmed sequence. Budding is mediated by adaptors that recruit cargoes and facilitate vesicle biogenesis. In *Saccharomyces cerevisiae*, the AP-3 adaptor complex directs cargoes from the Golgi to the lysosomal vacuole. The AP-3 core consists of small and medium subunits complexed with two non-identical large subunits, β3 (Apl6) and δ (Apl5). The C-termini of β3 and δ were thought to be flexible hinges linking the core to ear domains that bind accessory proteins involved in vesicular transport. We found by computational modeling that the yeast β3 and δ hinges are intrinsically disordered and lack folded ear domains. When either hinge is truncated, AP-3 is recruited to the Golgi, but vesicle budding is impaired and cargoes normally sorted into the AP-3 pathway are mistargeted. This budding deficiency causes AP-3 to accumulate on ring-like Golgi structures adjacent to GGA adaptors that, in wild-type cells, bud vesicles downstream of AP-3 during Golgi maturation. Thus, each of the disordered hinges of yeast AP-3 has a crucial role in mediating transport vesicle formation at the Golgi.

## Teaser

**Summary:** The AP-3 adaptor in *Saccharomyces cerevisiae* has intrinsically disordered regions required for AP-3 vesicle budding from the Golgi. Without these hinges, AP-3 remains associated with Golgi compartments as they mature.

## INTRODUCTION

Vesicle trafficking pathways ferry protein and lipid cargoes between different membrane-bound organelles in eukaryotic cells. This highly regulated process is orchestrated by cytosolic adaptor proteins specific to each trafficking pathway (reviewed in Robinson, 2015). Adaptor proteins capture and concentrate transmembrane proteins to be incorporated as cargoes of nascent transport vesicles. These adaptors also recruit other cytosolic proteins that facilitate vesicle budding and scission. In some cases, adaptors interact with proteins that mediate the fusion of vesicles with their target destination (reviewed in Angers and Merz, 2011). The diverse functions of vesicle adaptor proteins make them pivotal in maintaining cellular compartmentalization.

To accommodate the numerous different trafficking pathways within cells, a diversity of vesicle adaptors has evolved, including the family of adaptor protein (AP) complexes. Among the five characterized AP complexes, three (AP-1, AP-2 and AP-3) appear to be the most widespread throughout Eukarya (Hirst et al., 2011). Each AP complex functions in a distinct vesicle trafficking pathway. AP-2 is the best-characterized AP complex and is well known for its function in endocytosis, whereas AP-1 functions in vesicular transport between endosomes and the Golgi (Robinson, 2015; Casler et al., 2022; Robinson et al., 2024). AP-3 is the least-understood member among the conserved AP complexes, despite studies of AP-3 trafficking marking the advent of modern genetics (Morgan, 1910) and AP-3 deficiency resulting in various human pathologies (Horwitz et al., 2004; Dell'Angelica, 2009; Bowman et al., 2019). AP-3 mediates vesicular transport to lysosomes and lysosome-related organelles. In mammalian cells, AP-3 vesicles bud from endosomal or Golgi compartments (Peden et al., 2004; Theos et al., 2005). In yeast, AP-3 vesicles bud from the Golgi (Cowles et al., 1997a; Stepp et al., 1997). However, yeast cells have a minimal endomembrane system in which the Golgi performs some endosomal sorting functions (Day et al., 2018), so the AP-3 pathway is likely homologous in these different cellular systems.

Each AP complex is a heterotetramer consisting of a small subunit, a medium subunit and two large subunits that are non-identical but structurally similar. The quaternary structures of AP complexes are generally conserved: the N-terminal trunk domains of AP large subunits are compactly folded structures that assemble into heterotetramers with the medium and small subunits, forming the complex core. The core binds lipids that are specific to membrane microdomains and also interacts with peptide sequences in the cytosolic domains of transmembrane protein cargoes (reviewed in Kirchhausen et al., 2014). The AP-1 and AP-3 cores also bind Arf GTPases that facilitate the membrane recruitment of each complex (Stamnes and Rothman, 1993; Traub et al., 1993; Seaman et al., 1996; Ooi et al., 1998; Drake et al., 2000; Begley et al., 2024 preprint).

The C-terminus of each large subunit of mammalian AP-1 and AP-2 has a secondary structure known as an ear domain (also known as an appendage). Each ear connects to the core of the complex through an intrinsically disordered ‘hinge’ region consisting of 80–100 amino acids (Heuser and Keen, 1988). The AP-1 and AP-2 ears bind numerous accessory proteins that facilitate vesicle budding as well as other steps in the transport process (Owen et al., 1999; Traub et al., 1999; Praefcke et al., 2004; Ritter et al., 2004; Schmid et al., 2006; reviewed in Kirchhausen et al., 2014). Based on its functional homology, AP-3 is anticipated to interact similarly with accessory proteins via C-terminal ear domains that are linked by disordered hinge regions to one or both large subunits of the complex. Indeed, a handful of proteins that facilitate vesicular transport are known to bind the C-terminal regions of large subunits in AP-3, including Vps41, a member of the HOPS protein complex that tethers AP-3 vesicles at the target vacuole or lysosome membrane (Rehling et al., 1999; Angers and Merz, 2009; Cabrera et al., 2010; Asensio et al., 2013; Schoppe et al., 2020). However, in reconstructions of yeast and human AP-3 imaged by cryo-electron microscopy (EM), the C-termini of the large subunits were not resolved (Schoppe et al., 2021; Begley et al., 2024 preprint), so the extent of structural conservation in these regions of AP-3 is unknown.

Here, we present computational modeling suggesting that a folded ear domain is not present at the C-terminus of either large subunit of yeast AP-3. Instead, each C-terminal region is predicted to be entirely disordered. We investigated the role of each disordered hinge in yeast AP-3 by testing the effect of truncating each region. Our results show that hinge truncations do not disrupt the recruitment of AP-3 complexes to membranes, but each hinge is required for AP-3 vesicle budding from the Golgi. The consequences of AP-3 budding deficiency include Golgi enlargement and aberrant colocalization of AP-3 with GGA adaptors that normally function downstream of AP-3 during Golgi maturation, although GGA remains functional under these abnormal conditions.

## RESULTS

### Ear domain structures are absent from yeast AP-3 complexes in computational models

**Fig. 1.**
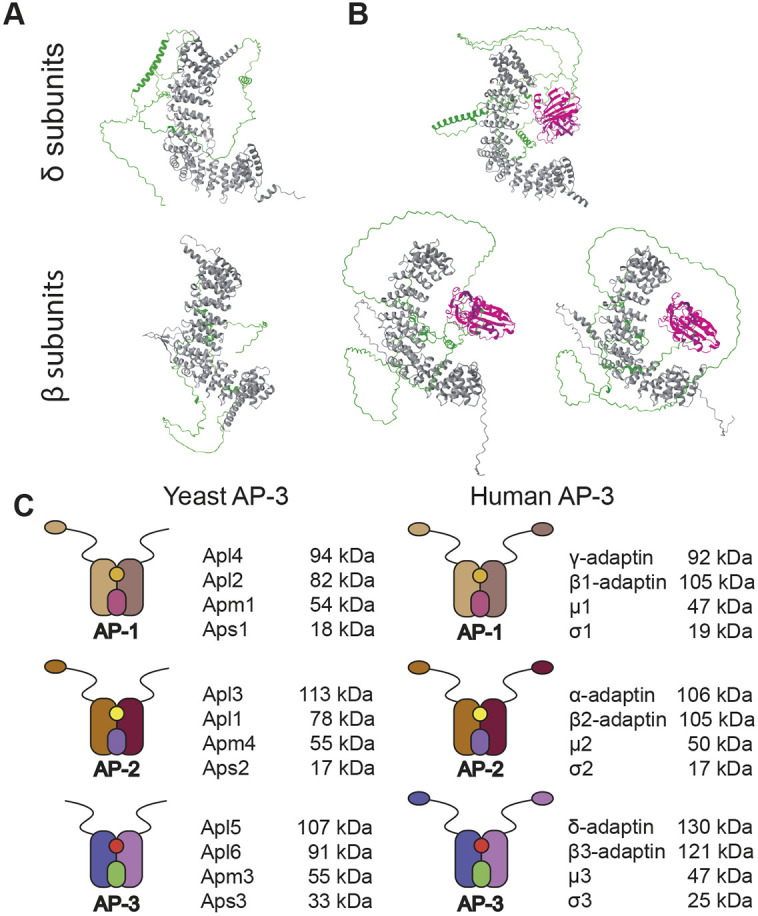
**Folded ear domains are predicted to be missing from yeast AP-3 complexes.** (A) Yeast AP-3 δ and β3 subunit structures predicted by AlphaFold2. (B) Human AP-3 δ, β3A and β3Β subunit structures predicted by AlphaFold2. In A,B, grey coloring indicates the trunk domains of each subunit, whereas green shows the hinge region. In B, magenta shows the structured ear domains on the C-terminus. (C) Cartoons of yeast versus human AP-1, AP-2 and AP-3 complexes based on computational modeling.

Cryo-EM analysis had shown that the yeast AP-3 core has a structure similar to the mammalian AP-1 and AP-2 core structures, although variations in flexibility were apparent between the complexes (Schoppe et al., 2021). Absent from the AP-3 structure was the C-terminal region of each large subunit, Apl5 and Apl6 (the δ and β3 subunits, respectively), suggesting that each region is disordered and does not contribute to the quaternary structure. Because of the overall structural and functional conservation among AP complexes, the C-terminus of each yeast AP-3 large subunit was expected to emanate from the core as an unstructured stretch of amino acids, with at least one of these regions linking to a folded ear domain (Edeling et al., 2006). However, computational modeling by AlphaFold2 (Fig. 1A) and Metapredict (Fig. S1A) indicate that the C-terminal regions of Apl5 and Apl6 lack ear domains and are, instead, mostly disordered, with no tertiary structure and only short regions of predicted α-helical secondary structure. To maintain consistency with nomenclature used for other AP complexes, the intrinsically disordered C-terminal regions of Apl5 and Apl6 are referred to here as hinges, although they do not appear to link the AP-3 core to folded ear domains as they do in AP-1 and AP-2.

Unlike yeast AP-3, the human AP-3 δ subunit (encoded by *AP3D1*) and each of the two human β3 subunit isoforms (β3A and β3B, encoded by *AP3B1* and *AP3B2*, respectively) are predicted by AlphaFold2 to have folded ear domains at the C-termini of their disordered hinges (Fig. 1B). The absence of ear domains in yeast δ and β3 raised the possibility that this secondary structure did not evolve in any of the AP complexes in *Saccharomyces cerevisiae*. However, in contrast to AP-3, AlphaFold2 does predict a folded ear domain at the C-terminus of the yeast AP-1 γ subunit (Apl4) and the AP-2 α subunit (Apl3), whereas ear domains are absent in β subunits of yeast AP-1 and AP-2 (Fig. S1B). Fig. 1C shows revised models comparing the AP-1, AP-2 and AP-3 complexes in *S. cerevisiae* and humans, based on the available crystallographic data and our computational modeling.

AP-3 in mammalian cells can function independently of clathrin (Zlatic et al., 2013), although direct binding of clathrin heavy chain to the human β3A subunit was detected *in vitro* (Dell'Angelica et al., 1998). This interaction depends on an amino acid sequence in the hinge of β3A that matches a conserved clathrin-binding motif (CBM; Dell'Angelica, 2001). The CBM consensus sequence (LΦ[polar]Φ[D/E]) is absent from Apl5 and Apl6 in yeast AP-3, although the Apl5 hinge has a CBM-like sequence (LLDLN) that lacks the negatively charged amino acid at position 5. We observed that clathrin heavy chain (Chc1) in yeast lysates was unable to bind the purified hinge regions of either Apl5 or Apl6 *in vitro*, whereas clathrin binding to the hinge of the AP-1 γ subunit (Apl4) was readily detected (Fig. S1C). This result agrees with prior work indicating that, in *S. cerevisiae*, clathrin does not bind AP-3 (Yeung et al., 1999) and AP-3 trafficking is unaffected by genetic mutation of clathrin (Vowels and Payne, 1998; Schoppe et al., 2020). Additionally, we found that deletion of the *APL5* or *APL6* genes did not exacerbate the growth defect of cells bearing the *chc1-521* mutation in clathrin heavy chain, unlike deletion of AP-1 genes in *chc1-521* mutant cells (Fig. S1D), which is also consistent with yeast AP-3 functioning independently of clathrin (Yeung et al., 1999).

### AP-3 trafficking in yeast requires the disordered hinge regions

**Fig. 2.**
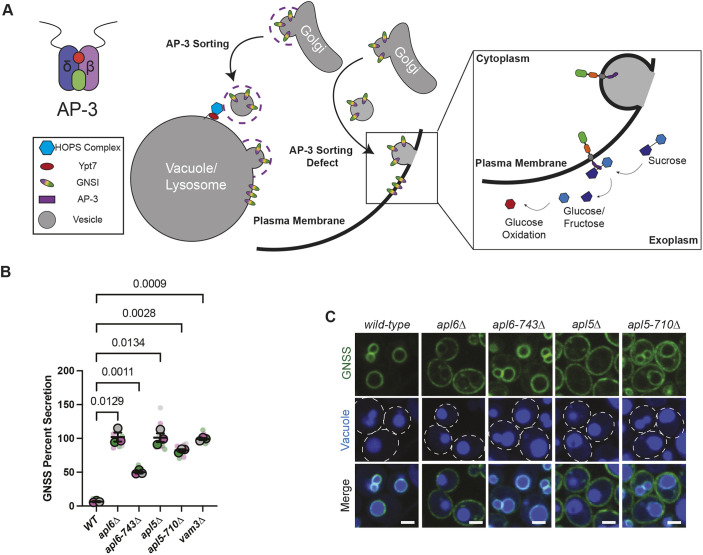
**AP-3 trafficking in yeast requires the hinge region of each AP-3 large subunit.** (A) Cartoon representation of the synthetic cargo GNSI (equivalent to GNSS) being sorted via the AP-3 pathway from the Golgi to the vacuole in wild-type yeast and missorted from the Golgi to the plasma membrane in mutant yeast with defects in the AP-3 pathway. (B) Comparative secretion of GNSS by liquid invertase assay. The data are representative of three independent experiments. Error bars represent the s.e.m. *P*-values were calculated using one-tailed paired Dunnett's *t*-test. (C) Microscopy showing the localization of GNSS in wild-type and AP-3-mutant cells. The vacuole lumen is stained with CMAC Blue and cell outlines are shown with white dotted lines. Images represent three independent experiments. Scale bars: 2 µm.

AP-3 mediates vesicular transport from the Golgi to the vacuole in yeast (Fig. 2A; Cowles et al., 1997a; Stepp et al., 1997). We investigated the extent to which this pathway requires the δ or β3 hinges by progressively truncating the C-terminus of Apl5 versus Apl6 (Fig. S2A). The effects of these truncations on AP-3 trafficking were evaluated using GNSS, a synthetic transmembrane protein cargo that serves as a qualitative and quantitative reporter of AP-3 sorting efficiency (Fig. 2A). GNSS is almost identical to GNSI, a synthetic AP-3 cargo we previously characterized (Plemel et al., 2021). Both GNSS and GNSI have green fluorescent protein (GFP) fused to the cytoplasmic domain of the v/R-SNARE Nyv1, which has a sorting signal that mediates entry into AP-3 vesicles budding from the Golgi (Reggiori et al., 2000). The transmembrane domain of GNSS and GNSI is derived from the v/R-SNARE Snc1, and the lumenal or exoplasmic domain of GNSS has two copies of the invertase enzyme encoded by the *SUC2* gene (Fig. 2A), whereas GNSI has one copy of invertase (Plemel et al., 2021). In wild-type cells, GNSS and GNSI are sorted from the Golgi to the vacuole membrane via the AP-3 pathway, but, in mutant cells lacking AP-3 function, these reporters are rerouted into the secretory pathway and delivered to the plasma membrane (Reggiori et al., 2000; Plemel et al., 2021).

Using a chromogenic assay that detects invertase activity at the cell surface (Darsow et al., 2000; Plemel et al., 2021), we observed mistargeting of GNSS to the plasma membrane in mutant strains lacking either Apl5 (*apl5*Δ) or Apl6 (*apl6*Δ) but not in wild-type yeast or in *apl5*Δ or *apl6*Δ cells harboring plasmids encoding the wild-type *APL5* or *APL6* gene (Fig. S2B). Deletion-mutant strains transformed with the series of plasmid-borne *apl5* or *apl6* truncation alleles showed partial to complete loss of GNSS sorting (Fig. S2B). We further analyzed *apl5-710*Δ and *apl6-743*Δ truncation-mutant alleles because of their effects on AP-3 localization, as described below. The extent to which *apl5-710*Δ and *apl6-743*Δ caused rerouting of the GNSS cargo into the secretory pathway was quantified by measuring the fraction of total cellular invertase activity at the plasma membrane of cells grown in liquid culture. We found that ∼80% of GNSS was missorted in *apl5-710*Δ cells, whereas ∼50% of this AP-3 cargo was missorted in *apl6-743*Δ cells (Fig. 2B). These values are consistent with our microscopic analyses, which showed increased GNSS fluorescence at the plasma membrane of *apl5-710*Δ cells compared to that in *apl6-743*Δ cells, whereas in wild-type cells, virtually all of the GNSS fluorescence was at the vacuole membrane (Fig. 2C). Thus, efficient cargo trafficking via the AP-3 pathway depends on the disordered hinges of the two AP-3 large subunits.

### Truncation of either hinge region causes AP-3 accumulation at the Golgi

Having established that yeast AP-3 cargo sorting is disrupted by truncating the disordered hinge region of either the AP-3 β3 (Apl6) or δ (Apl5) subunits, we investigated the effects of these truncations on the intracellular localization of the AP-3 complex. We constructed *APL5–GFP* and *APL6–GFP* fusions that were chromosomally integrated and fully functional, based on GNSS sorting assays (Fig. S3A). In wild-type cells, these GFP fusions were previously shown to localize in close proximity to Sec7 (Day et al., 2018). Sec7 is a marker of the trans-Golgi network (TGN) (e.g. Daboussi et al., 2012; Day et al., 2018; Franzusoff et al., 1991), where it functions as a guanine nucleotide exchange factor (GEF) for the redundant Arf1 and Arf2 GTPases in *S. cerevisiae* (Sata et al., 1998; Losev et al., 2006; Casanova, 2007). Using confocal fluorescence microscopy, we examined the localization of Apl5–GFP or Apl6–GFP relative to Sec7 fused to the MARS red fluorescent protein in yeast expressing wild-type or truncated *apl6* or *apl5* alleles (unpublished observations, M.L. and G.O.). In this analysis, we found that the *apl6-743*Δ and *apl5-710*Δ truncations caused AP-3 (labeled by Apl5–GFP or Apl6–GFP, respectively) to accumulate at enlarged structures that were round and sometimes hollow, reminiscent of rings or donuts. For this reason, we focused our studies on understanding the effects of these specific truncation-mutant alleles.

**Fig. 3.**
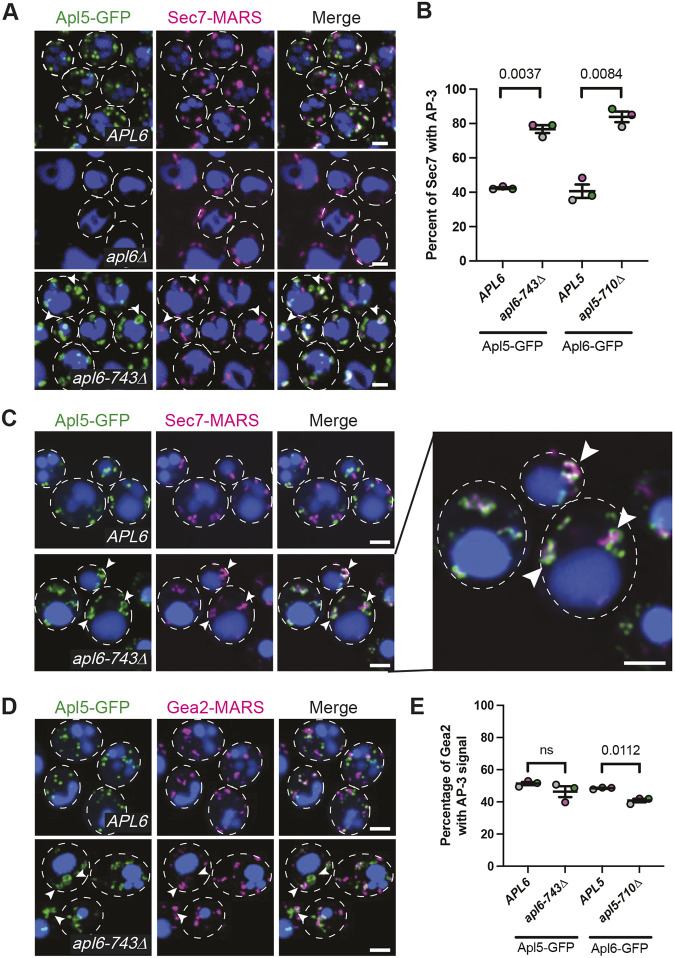
**Hinge truncations inhibit AP-3 vesicle budding from the Golgi.** (A) Conventional confocal fluorescence microscopy showing GFP-tagged AP-3 localization relative to Sec7–MARS. (B) Quantitation of the percentage of Sec7 colocalizing with AP-3 large subunits in wild-type cells and truncation mutants in images obtained by super-resolution confocal fluorescence microscopy. (C) Super-resolution confocal fluorescence microscopy showing the distribution of AP-3 relative to Sec7; arrowheads indicate round structures. Right: zoomed-in image of AP-3 and Sec7 in an AP-3 hinge truncation-mutant cell. (D) Super-resolution confocal fluorescence microscopy showing the distribution of AP-3 relative to Gea2; arrowheads indicate round structures. In A,C,D, the vacuole lumen was stained with CMAC Blue and cell outlines are shown with white dotted lines. Scale bars: 2 µm. (E) Quantitation of the percentage of Gea2 colocalizing with AP-3 large subunits in wild-type and AP-3 truncation-mutant cells in images obtained by super-resolution confocal fluorescence microscopy. In B,E, each point represents the average of at least 200 cells, error bars represent the s.e.m. of three independent experiments, and *P*-values were determined by two-tailed paired Student's *t*-test.

In wild-type cells observed by conventional confocal fluorescence microscopy, we found numerous Apl5–GFP puncta, many of which were in close proximity to puncta labeled by Sec7–MARS (Fig. 3A). We scored ∼45% of Sec7–MARS compartments as colocalizing with either Apl5–GFP or Apl6–GFP (Fig. 3B). Our observation that <50% of Sec7–MARS colocalizes with AP-3 agrees with work showing that AP-3 is recruited to maturing Golgi cisternae upstream of Sec7 (Day et al., 2018; Tojima et al., 2019; Highland and Fromme, 2021). In *apl6-743*Δ cells, we observed Apl5–GFP puncta that were substantially enlarged compared to Apl5–GFP puncta in wild-type *APL6* cells (Fig. 3A). Moreover, the fraction of Sec7–MARS overlapping Apl5–GFP almost doubled in response to Apl6 truncation (Fig. 3B). We observed a similar localization pattern for Apl6–GFP: ∼40% of Sec7–MARS puncta colocalized with Apl6–GFP in wild-type *APL5* cells, whereas in *apl5-710*Δ cells, Apl6–GFP puncta were enlarged (Fig. S3B) and the percentage of overlapping Sec7–MARS puncta increased more than twofold (Fig. 3B). Therefore, truncation of the hinge region of either AP-3 large subunit causes the AP-3 complex to accumulate at TGN compartments marked by Sec7. A plausible interpretation of these findings is that the hinge regions are needed for successful completion of AP-3 budding from the Golgi but not for AP-3 recruitment to Golgi membranes. As expected, Apl5–GFP puncta were not seen in *apl6*Δ cells (Fig. 3A) and Apl6–GFP puncta were not observed in *apl5*Δ cells (Fig. S3B). Moreover, neither of these GFP fusions was localized to puncta in deletion-mutant strains lacking either the *APM3* or *APS3* gene that encodes the medium or small AP-3 subunit, respectively (unpublished observations, M.L. and G.O.). All four subunits, therefore, are required for efficient recruitment of AP-3 complexes to the Golgi.

In the above analyses, we occasionally observed Apl5–GFP or Apl6–GFP in a circular, donut-shaped pattern surrounding Sec7–MARS, but only in mutant cells bearing a hinge truncation and not in cells expressing full-length Apl6 and Apl5 (arrowheads in Fig. 3A and Fig. S3B). Using a spinning disk confocal microscope equipped for super-resolution imaging, puncta of GFP-tagged AP-3 surrounding Sec7–MARS were more clearly evident and appeared as segmented radial structures with interspersed microdomains of AP-3 (arrowheads in Fig. 3C and in Fig. S3C). Additionally, Sec7-positive structures were more clearly resolved by super-resolution imaging than they were in our conventional confocal fluorescence images and showed a range of morphologies, with the majority seen as oblong cisterna-like structures and a minority observed as crescents or fully rounded structures (Fig. S3D). Clusters of AP-3 with Sec7 were present in >90% of *apl6-743*Δ and *apl5-710*Δ mutant cells but were rarely observed (∼5%) in wild-type cells expressing full-length AP-3 subunits (Fig. 3C; *n*≥200). The sharp increase in clusters of AP-3 and Sec7 fluorescence again indicates that AP-3 aberrantly accumulates on TGN compartments when the disordered hinge of either AP-3 large subunit is truncated.

Sec7 is one of three GEFs that act in a spatiotemporal sequence to activate the Arf1 and Arf2 GTPases during Golgi maturation. Upstream of Sec7 at the TGN, Arf1 and/or Arf2 activation is catalyzed by Gea1 at the early Golgi, followed by Gea2 at the medial and late Golgi (Peyroche et al., 1996; Spang et al., 2001; Gustafson and Fromme, 2017). Activated (GTP-bound) Arf1 and Arf2 recruit diverse proteins, including vesicle adaptors (Goldberg, 1999; Hirst et al., 2000; Boman et al., 2000; Dell'Angelica et al., 2000; Puertollano et al., 2001). Sequential activation of Arf1 and Arf2, in combination with other signals, is thought to result in waves of vesicle budding from Golgi compartments as they mature from cis- to medial- to late-Golgi and the TGN (Bui et al., 2009; Highland and Fromme, 2021).

To determine whether AP-3 hinge truncations affect AP-3 recruitment to Golgi compartments upstream of Sec7, we examined the localization of Apl5–GFP or Apl6–GFP in cells expressing a Gea2–MARS fusion protein. In wild-type cells, we observed Gea2–MARS colocalizing with GFP-tagged AP-3 subunits at a frequency similar to that of Sec7–MARS colocalization with AP-3. However, Gea2–MARS colocalization with AP-3 was modestly decreased in cells expressing AP-3 hinge truncations (Fig. 3D,E). Together with the increase in AP-3 and Sec7–MARS colocalization that we observed in AP-3 hinge truncation mutants (Fig. 3B), this result suggests that hinge truncations cause aberrant retention of AP-3 at Golgi compartments undergoing medial- to late-Golgi/TGN maturation. Notably, a range of Gea2–MARS localization patterns was observed in AP-3 hinge truncation mutants compared to those seen in wild-type cells. Among the patterns of Gea2–MARS localization seen in hinge truncation mutants were donuts that were directly adjacent to Apl5–GFP or Apl6–GFP donuts, indicating that, when AP-3 vesicle budding is blocked, AP-3 accumulates at membranes distinct from compartments containing Gea2 (arrowheads in Fig. 3D and Fig. S3E,F).

### Hinge truncations inhibit AP-3 vesicle budding

Based on the shift of AP-3 toward Sec7-marked TGN compartments in mutant cells expressing AP-3 hinge truncations, we further examined the spatial relationship between AP-3 and Sec7 by high-speed time-lapse microscopy. Samples were illuminated using variable angle epifluorescence (VAEF) microscopy to decrease photodamage (Konopka and Bednarek, 2008), and fluorescence emission signals were split so that paired images of GFP-tagged AP-3 and Sec7–MARS could be captured simultaneously. Analyses of 30-s movies acquired at 20 frames per second revealed two populations of Apl5–GFP puncta in wild-type *APL6* cells. The first set consisted of puncta that were relatively static and often colocalized with Sec7–MARS; the second set consisted of Apl5–GFP puncta that were extremely dynamic, moving away and sometimes toward the more static population of Apl5–GFP and Sec7–MARS puncta (see Movie 1). In marked contrast to wild-type *APL6* cells, the dynamic population of Apl5–GFP puncta was largely or completely missing in *apl6-743*Δ cells; instead, almost all Apl5–GFP puncta exhibited constrained movement when paired with the *apl6-743*Δ mutation, often orbiting Sec7–MARS, rather than moving away (see Movie 2). The relatively immobile population of Apl5–GFP and Sec7–MARS puncta corresponds to the clusters of AP-3 and Sec7 in three-dimensional image stacks that we observed by confocal microscopy at much lower frame rates (Fig. 3A,C).

**Fig. 4.**
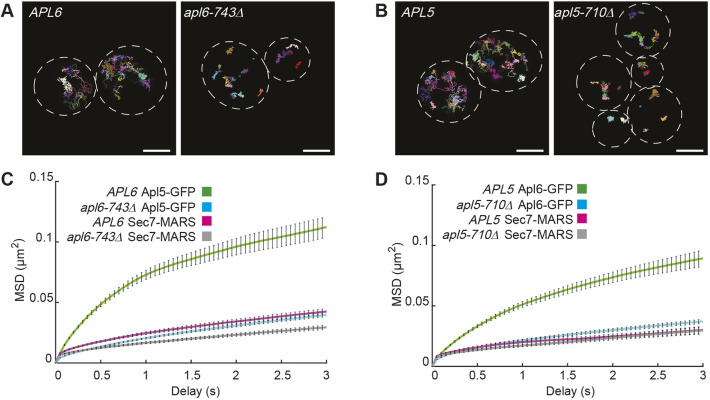
**Hinge truncations inhibit AP-3 vesicle budding.** (A,B) Track maps of a 30-s movie with 50-ms exposure times, tracking the mobility of Sec7–MARS and either Apl5–GFP (A) or Apl6–GFP (B) in the indicated strains. Scale bars: 2 µm. (C,D) Mean squared displacement (MSD) values over 3 s for either (C) Apl5–GFP and Sec7–MARS in yeast expressing either *APL6* or *apl6-743*Δ, or (D) Apl6–GFP and Sec7–MARS in cells expressing either *APL5* or *apl5-710*Δ. At least 50 cells were quantified in each group across nine motility assays such as those depicted in A and B. Movies were captured on three different days. Error bars represent s.e.m. for puncta displacement at that time.

Tracking individual particles in 30-s movies demonstrated the dramatic difference in the mobility of Apl5–GFP in wild-type *APL6* versus *apl6-743*Δ mutant cells. Examples are shown for single cells in Fig. 4A. Particle displacements were quantitated from cell populations in Fig. 4C. A similar pattern of movement was observed for Apl6–GFP, which exhibited high mobility in wild-type *APL5* cells but showed restricted movement in *apl5-710*Δ mutant cells (Fig. 4B,D; see Movies 3 and 4). The restricted mobility of AP-3 puncta and accumulation of AP-3 at Sec7-positive Golgi resulting from AP-3 hinge truncations suggest that AP-3 vesicle budding from the Golgi requires the disordered hinge of each large subunit. We infer that the slow-moving AP-3 puncta in wild-type cells are incipient vesicles forming at the Golgi, whereas the fast-moving puncta are free, post-Golgi AP-3 vesicles that have not yet docked at the vacuole target membrane. The loss of fast-moving AP-3 puncta and retention of AP-3 at Sec7-positive Golgi in hinge truncation mutants support working models in which each AP-3 hinge is needed for vesicle budding. This interpretation is buttressed by previous work showing that AP-3 does not rapidly uncoat upon budding. Instead, AP-3 remains associated with the transport vesicle until the vesicle has docked or fused at the vacuole membrane (Angers and Merz, 2011; Schwartz et al., 2017; Schoppe et al., 2020).

**Fig. 5.**
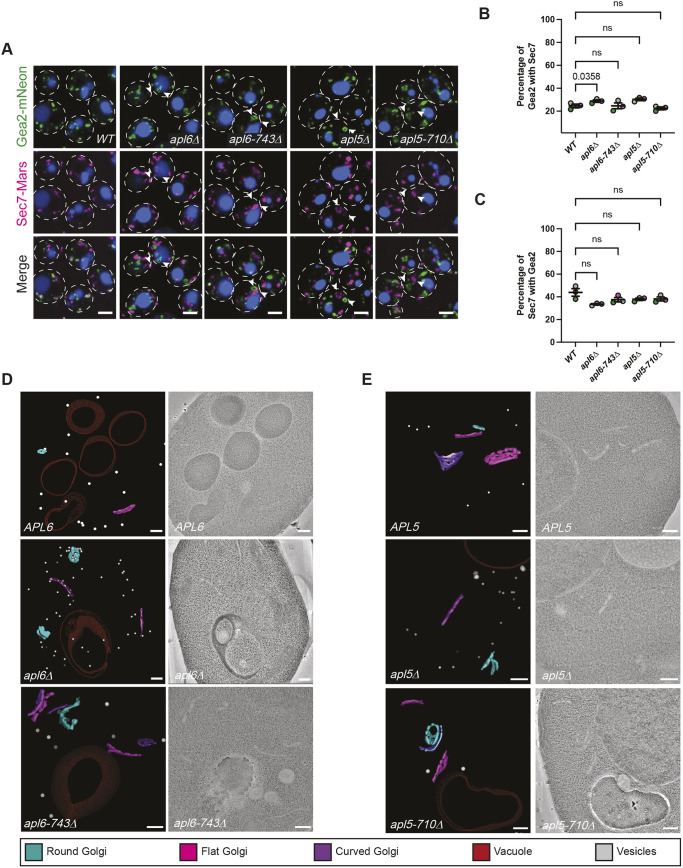
**AP-3 dysfunction alters Golgi morphology.** (A) Super-resolution confocal fluorescence microscopy showing the effects of AP-3 mutations on the relative localizations of Gea2–mNeon and Sec7–MARS. Scale bars: 2 µm. (B,C) Graphs showing (B) the percentage of overlap of Gea2 puncta with Sec7, and (C) the percent of overlap of Sec7 with Gea2. Error bars show s.e.m. and *P*-values were determined by one-tailed paired Dunett's test. (D,E) Tomographic representatives of Golgi morphology changes in wild-type versus *apl6-* or *apl5-*mutant cells. Flat Golgi are shown in magenta, round Golgi in cyan, curved Golgi in purple, vacuole membranes in red and cytosolic vesicles in grey. Scale bars: 200 nm.

Based on the changes observed in Gea2 and Sec7 localization resulting from AP-3 hinge truncations (Fig. 3), we investigated the localization of these Arf GEFs relative to each other by super-resolution microscopy. In wild-type cells, Gea2–mNeon and Sec7–MARS rarely overlapped (Fig. 5A–C), consistent with previous observations indicating that Golgi compartments are sequentially marked by each GEF (Bui et al., 2009; Highland and Fromme, 2021). When either hinge of AP-3 was truncated, spatial separation between Gea2–mNeon and Sec7–MARS was maintained (Fig. 5B,C), but each exhibited radial patterning. This patterning was unlike the punctate or tubular shapes observed in wild-type cells, but similar to what was observed for each GEF when co-expressed with GFP-tagged AP-3 in *apl6* or *apl5* truncation-mutant cells (Fig. 3). Despite their radial distributions, no substantial differences were seen in the size or number of Gea2–mNeon or Sec7–MARS puncta in truncation-mutant cells compared to those in wild-type cells. However, there was a slight difference in *apl5*Δ cells that had larger puncta of Sec7–MARS, which likely represent donuts for which the centers were not resolved by light microscopy (Fig. S4A–D).

The changes in the localization patterns of Gea2 and Sec7 prompted us to investigate whether AP-3 hinge truncations altered Golgi morphology, which we examined at higher resolution using electron tomography. In tomographic reconstructions of wild-type *S. cerevisiae,* Golgi cisternae appear as elongated, fenestrated flat disk-shaped compartments or as curved or rounded compartments (Fig. 5D,E; see Movie 5). When measuring the total surface area of these Golgi membranes in wild-type cells, flat Golgi were consistently larger than their rounded counterparts (Fig. S4E,F). The membrane surface areas of flat Golgi remained consistent when AP-3 hinges were truncated or when AP-3 large subunits were deleted (Fig. S4E,F). AP-3 large subunit deletions and hinge truncations caused some rounded Golgi to more than double their total membrane surface areas, although this effect was not statistically significant (Fig. S4E,F; see Movies 6 and 7). Immunogold labeling of thin sections indicated that Apl5–GFP and Apl6–GFP localized to both flat and rounded compartments in wild-type cells. In contrast, AP-3 hinge truncations resulted in twice as much immunogold labeling on rounded Golgi relative to that on flat Golgi (Fig. S4G–J), suggesting that unbudded AP-3 complexes prefer curved Golgi membranes, although more direct experimentation will be needed to determine the extent to which AP-3 has this property.

### Budding-defective AP-3 commingles with downstream Golgi vesicle adaptors

Studies of the temporal and spatial dynamics of transport vesicle adaptors in yeast indicate that AP-3 is recruited to the Golgi prior to two other adaptors, GGA and AP-1. GGA adaptors (Gga1 and Gga2) mediate vesicular transport from the TGN to late endosomes (Black and Pelham, 2000; Dell'Angelica et al., 2000; Hirst et al., 2000; Zhdankina et al., 2001); AP-1 mediates recycling from TGN or early endosomal compartments to earlier Golgi cisternae (Daboussi et al., 2012; Day et al., 2018; Casler et al., 2019). GGA recruitment to the TGN coincides with Sec7 arrival, both spatially and temporally, and precedes AP-1 recruitment to Sec7-positive compartments (Daboussi et al., 2012; Day et al., 2018; Casler and Glick, 2020; Tojima et al., 2019). Further supporting this spatiotemporal sequence, genetic studies have shown that sorting into the AP-3 pathway occurs upstream of GGA sorting (Cowles et al., 1997b). Because AP-3 hinge truncations cause accumulation of AP-3 at Sec7 compartments (Fig. 3), we investigated the extent to which hinge truncations affect the localization of AP-3 relative to GGA and AP-1.

**Fig. 6.**
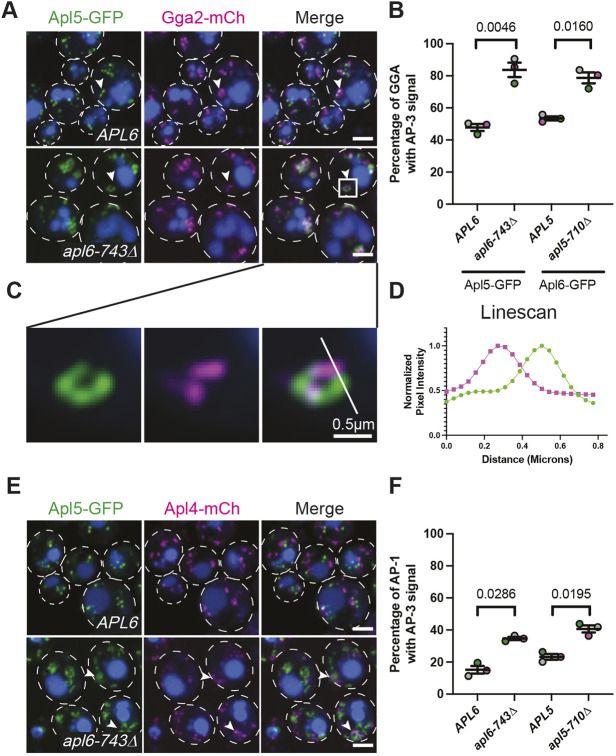
**Budding deficiency causes AP-3 to accumulate at late Golgi compartments.** (A) Super-resolution confocal fluorescence microscopy showing the distribution of AP-3 relative to Gga2. Arrowheads indicate rounded structures for both Gga2 and AP-3. (B) Quantitation of the percentage of Gga2 colocalizing with AP-3 large subunits in wild-type AP-3 and truncation-mutant AP-3 cells. (C) Zoomed-in image of AP-3 and GGA commingling donuts. Scale bar: 0.5 µm. (D) Line scan of AP-3 and GGA in an AP-3 truncation mutant. (E) Super-resolution confocal fluorescence microscopy showing the distribution of AP-3 relative to AP-1. Arrowheads indicate rounded structures for both AP-1 and AP-3. In A,E, the vacuole lumen was stained with CMAC Blue and cell outlines are shown with white dotted lines. Scale bars: 2 µm. (F) Quantitation of the percentage of AP-1 colocalizing with AP-3 large subunits in wild-type AP-3 and truncation-mutant AP-3 cells. In B,F, each point represents the average of at least 200 cells, error bars show the s.e.m. and *P*-values were determined by two-tailed paired Student's *t*-test.

In wild-type cells, mCherry-tagged GGA (Gga2–mCh) was frequently observed as clusters of puncta arranged in a circular array (Fig. 6A; Fig. S5A). These radial arrangements resembled the donut-shaped clusters of AP-3 puncta in cells in which Apl5 or Apl6 had been truncated (Fig. 3; Figs S3, S4). By super-resolution microscopy, an individual Apl5–GFP punctum was seen occasionally inserted between the radial Gga2–mCh puncta, but the majority of Apl5–GFP puncta were spatially separated from Gga2–mCh in wild-type cells (Fig. 6A). In *apl6-743*Δ cells, however, puncta marked by Gga2–mCh versus Apl5–GFP were frequently seen alternating in ring-like structures but with little direct overlap (Fig. 6B–D). Similar spatial relationships between Apl6–GFP and Gga2–mCh were seen in wild-type *APL5* versus mutant *apl5-710*Δ cells (Fig. S5A,B). Taken together, these results indicate that budding-deficient AP-3 complexes accumulate and are retained on Golgi compartments to which Gga2 is recruited, but each adaptor preferentially occupies distinct sub-micrometer membrane domains.

Unlike Gga2–mCh, AP-1 (labeled by Apl4–mCh) was predominantly observed in wild-type *APL6* cells as unorganized clusters or distinct puncta that were more spatially separated from Apl5–GFP puncta (Fig. 6E). Concurrently, the extent of overlap between Apl4–mCh and Apl5–GFP was considerably less than the overlap observed between Apl5–GFP and Gga2–mCh (Fig. 6F). In *apl6-743*Δ cells, Apl4–mCh puncta remained mostly separate from the radial clusters of Apl5–GFP puncta (Fig. 6E, lower panel; Fig. 6F), although the frequency of overlap doubled and Apl4–mCh puncta were occasionally seen intertwined with Apl5–GFP puncta (arrowheads in Fig. 6E). A similar pattern was observed in wild-type *APL5* versus *apl5-710*Δ cells expressing Apl6–GFP with Apl4–mCh (Fig. 6F; Fig. S5C). Collectively, these observations indicate that the AP-3 budding defect caused by hinge truncations results in AP-3 retention at late Golgi or TGN compartments as they mature, with AP-3 accumulating predominantly alongside GGA adaptors and some fraction of AP-3 persisting at the Golgi until AP-1 is recruited.

### AP-3 dysfunction does not prevent GGA sorting activity

The data described above indicate that budding-defective AP-3 persists at Golgi cisternae as these compartments mature and acquire GGA and AP-1 vesicle adaptors, which normally operate downstream of AP-3. Under these conditions, AP-3 aberrantly colocalizes most strongly with Gga2, which (with its redundant paralog, Gga1) functions to sort transmembrane proteins into the vacuolar protein sorting (VPS) pathway to deliver cargoes to prevacuolar endosomes *en route* to the vacuole. The best characterized cargo of the VPS pathway is Vps10, a transmembrane receptor for the soluble vacuolar hydrolase carboxypeptidase Y (CPY; Marcusson et al., 1994). After binding newly synthesized CPY in the Golgi, Vps10 is sorted by Gga1 or Gga2 into vesicles that bud from the TGN and fuse with the prevacuolar endosome, where Vps10 releases CPY and is subsequently retrieved to the Golgi through the retromer pathway (Seaman et al., 1998).

**Fig. 7.**
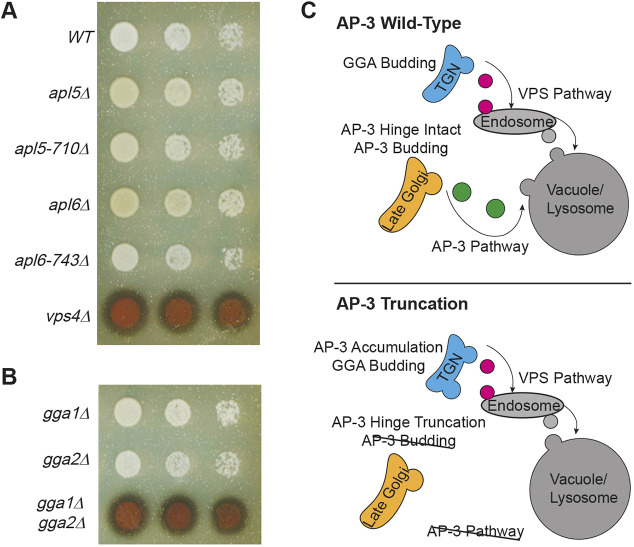
**AP-3 dysfunction does not affect GGA sorting activity.** (A,B) Invertase overlay investigating the effects of AP-3 mutations (A) versus GGA mutations (B) on the sorting of CPY invertase, which follows the vacuolar protein sorting (VPS) pathway. Each overlay reaction was imaged after 30 min. Cellular concentrations for each strain were 1.0, 0.1 and 0.01 OD_600_ units/ml (left to right). Images represent three independent experiments. (C) Cartoon illustrating the departure of AP-3 vesicles at the late Golgi and GGA vesicle departure at the trans-Golgi network (TGN). AP-3 cargoes follow the AP-3 pathway directly to the vacuole, whereas GGA cargoes follow the VPS pathway (upper panel). When AP-3 hinges are truncated, AP-3 accumulates onto the TGN and AP-3 cargo sorting is perturbed, whereas VPS cargo sorting functions normally (lower panel).

Deletion of AP-3 subunit genes does not affect CPY sorting (Cowles et al., 1997a; Stepp et al., 1997). Likewise, genetic disruption of the VPS pathway does not affect the sorting of AP-3 cargoes (Cowles et al., 1997b). Thus, the AP-3 and VPS pathways have been considered to be mechanistically distinct. However, because of the abnormal retention of budding-defective AP-3 at Golgi compartments to which GGA adaptors are recruited (Fig. 6C), we investigated whether GGA function is affected by the AP-3 hinge truncations. For this analysis, we used the CPY-invertase fusion protein that has been used extensively to study the VPS pathway (Bankaitis et al., 1986). As in our analyses of GNSS sorting (Fig. 2B), we evaluated CPY-invertase sorting in cells growing on agar medium using the chromogenic assay that detects secreted invertase activity (Darsow et al., 2000). Wild-type cells efficiently sort CPY-invertase to the vacuole and, thus, colonies appear white in this assay, but mutations that disrupt Vps10 trafficking between the TGN and prevacuolar endosome (e.g. *vps4*Δ) cause aberrant secretion of newly synthesized CPY-invertase, resulting in darkened colonies (Fig. 7A,B). CPY-invertase secretion was minimal upon deletion of *APL5* or *APL6*, consistent with earlier work (Cowles et al., 1997a; Stepp et al., 1997). Similarly, no defect in CPY sorting was detected in *apl5-710*Δ or *apl6-743*Δ cells (Fig. 7A,B). Thus, the aberrant retention of AP-3 at Golgi compartments does not impede GGA-mediated sorting (Fig. 7C).

## DISCUSSION

Prior studies have shown that the C-termini of mammalian AP-1 and AP-2 large subunits consist of unstructured amino acid sequences that act as hinges linking the core of each complex to ear domain secondary structures. Ear domains bind diverse accessory proteins that facilitate different stages of vesicular transport (reviewed in Kirchhausen et al., 2014; Robinson, 2015). Because of their central role in AP-1 and AP-2 function, we were surprised to find that computational analyses predicted that neither the β3 subunit (Apl6) nor the δ subunit (Apl5) of yeast AP-3 has an ear domain, although both subunits are predicted to have hinges that are mostly disordered, similar to those observed for AP-1 and AP-2 hinges. Phylogenetic studies have also suggested that ear domains are absent from β subunits in the AP-1, AP-2 and AP-3 complexes in the majority of fungal species (Martzoukou et al., 2017). Nonetheless, ear domains appear to have evolved in *S. cerevisiae* (and potentially in other fungi), based on computational models predicting an ear domain near the C-terminal end of an unstructured hinge in the γ subunit of AP-1 (Apl4) and the α subunit of AP-2 (Apl3). Why ear domains are not uniformly present in AP complexes is unclear.

Despite lacking folded ear domains, our experiments show that the hinges of Apl6 (β3) and Apl5 (δ) are required for AP-3 function in yeast. Truncation of either hinge disrupts cargo trafficking from the Golgi to the vacuole via the AP-3 pathway while having no apparent effect on AP-3 complex assembly, based on mass spectrometry of immuno-isolated AP-3 complexes from yeast cells expressing truncated subunits (unpublished results, C.G.A. and A.J.M.). Hinge truncations also do not disrupt recruitment of AP-3 to the Golgi (Fig. 3). Instead, each truncation inhibits AP-3 vesicle budding, resulting in the accumulation of AP-3 at or near TGN compartments marked by Sec7. We found no evidence that unbudded AP-3 complexes trap cargoes at the Golgi, as neither the *apl6-743*Δ truncation nor the *apl5-710*Δ truncation caused GNSS cargo fluorescence to accumulate at intracellular puncta (Fig. 2). Likewise, the *apl6-743*Δ truncation did not enhance the recovery of cargoes with immuno-isolated AP-3 complexes that were analyzed by mass spectrometry or by western blotting (unpublished results, M.L., C.G.A., A.J.M. and G.O.), although we cannot rule out the possibility that cargo accumulations might be detected using other methods. Similar to truncation of the yeast AP-3 δ or β3 subunits, C-terminal truncation of the mouse β3A subunit was found to disable AP-3 function but not its recruitment to perinuclear (Golgi or endosomal) compartments in cultured cells (Peden et al., 2002), although this earlier study did not directly determine whether mouse β3A truncation affected the budding of AP-3 vesicles. Curiously, cargo endocytosis in HeLa cells was unaffected by truncation of the AP-2 α2 subunit or by mutations in accessory-binding sites that had been identified in this subunit (Motley et al., 2006), although α2 truncation altered the kinetics of endocytic vesicle formation and disrupted events downstream of vesicle budding (Aguet et al., 2013; Reis et al., 2015).

Our observation that truncating either AP-3 hinge disables AP-3 vesicle budding was most vividly revealed by high-speed time-lapse fluorescence microscopy, which showed that truncated AP-3 is relatively immobile compared to wild-type AP-3. This disparity in motion raises the possibility that the unstructured hinges of AP-3 have a direct role in membrane remodeling and fission, as has been observed in other proteins with intrinsically disordered regions (Snead and Stachowiak, 2018). Another possibility is that the biophysical properties of the disordered hinges control the mobility and/or orientation of AP-3 complexes at the membrane surface, as has been seen for the disordered N-terminus of oxysterol-binding protein at membrane contact sites between the endoplasmic reticulum and the TGN (Jamecna et al., 2019). These hypotheses are not mutually exclusive with one another or with the possibility that the hinges of Apl5 and Apl6 might recruit other proteins that facilitate AP-3 vesicle budding, as observed for other AP complexes (Kirchhausen et al., 2014). For example, the hinges of AP-1 and AP-2 large subunits interact with clathrin coat proteins. In the case of yeast AP-3, however, clathrin is an unlikely partner because cargo sorting via the AP-3 pathway in *S. cerevisiae* is unaffected by mutation of the clathrin heavy or light chain genes (Vowels and Payne, 1998; Schoppe et al., 2020). Clathrin function is also dispensable for AP-3 vesicle budding in cultured rat cells (Zlatic et al., 2013), and AP-3 subunits do not copurify with clathrin-coated vesicles isolated from bovine brain (Newman et al., 1995; Simpson et al., 1996; Dell'Angelica et al., 1997). The ability of AP-3 to operate independently of clathrin might be explained by phylogenetic analyses suggesting that AP-3 is the most ancient member of the AP family (Hirst et al., 2011).

Understanding the mechanisms of AP-3 function would benefit from the identification of accessory proteins that interact with the hinges of the β3 and δ subunits. Such interaction partners are expected to include factors that facilitate multiple steps in AP-3 vesicle trafficking, like many of the proteins that interact with the hinge or ear domains of AP-1 and AP-2 large subunits (Kirchhausen et al., 2014). Although relatively few AP-3-binding partners have been identified thus far, a well-characterized interactor is Vps41, which binds the hinge of Apl5 in yeast (Rehling et al., 1999). Vps41 interaction with AP-3 is conserved in mammalian cells (Salazar et al., 2009), and earlier studies speculated that Vps41 replaces clathrin as an outer-shell coat protein for AP-3 vesicles (Rehling et al., 1999; Darsow et al., 2001; Asensio et al., 2013). However, Vps41 was subsequently characterized as a subunit of the homotypic fusion and vacuole protein sorting (HOPS) membrane tethering complex (Seals et al., 2000), and later studies established that it is in the context of HOPS in which Vps41 interacts with Apl5, serving to capture AP-3 vesicles at the vacuole or lysosome rather than facilitate AP-3 vesicle budding (Angers and Merz, 2009; Schoppe et al., 2020).

The Vps41 binding site in AP-3 is located in the hinge sequence that was deleted in *apl5-710*Δ. Although our results clearly show that Apl5 truncation inhibits AP-3 vesicle budding from the Golgi, defective AP-3 cargo sorting also occurs upon genetic disruption of membrane fusion at the vacuole (Schwartz et al., 2017; Plemel et al., 2021). This fusion step relies on HOPS-mediated tethering of AP-3 vesicles to the vacuole membrane (Angers and Merz, 2009). The defect in AP-3 vesicle budding combined with the loss of Vps41 binding to AP-3 in *apl5-710*Δ cells could explain why this mutant strain has a stronger cargo-missorting phenotype (∼80% missorted GNSS) compared to *apl6-743*Δ cells (∼50% missorted GNSS), although testing this hypothesis would require creative restoration of the Vps41-binding site in cells expressing the truncated Apl5 protein. In either case, the different severity to which truncation of Apl5 versus Apl6 affects AP-3 cargo sorting suggests that the hinge regions are not functionally redundant, even though truncation of either hinge impairs the budding of AP-3 vesicles. We anticipate that the Apl5 and Apl6 hinges bind distinct accessory proteins involved in AP-3 vesicle trafficking, although some interaction partners might be shared, as has been observed for hinge ear regions of other AP complexes (Traub, 2005).

A search for regulators of AP-3 vesicle trafficking in yeast was carried out by proximity-dependent biotinylation using Apl5 fused at its C-terminus to a biotin ligase (Schoppe et al., 2020). This approach resulted in the identification of Age2, one of several GTPase-accelerating proteins (GAPs) that function to inactivate Arf GTPases (Poon et al., 2001). In their active GTP-bound state, Arfs associate with Golgi membranes and facilitate the recruitment of AP-3 (Ooi et al., 1998; Drake et al., 2000) and other Golgi vesicle adaptors (reviewed in D'Souza-Schorey and Chavrier, 2006). In *S. cerevisiae*, the redundant Arf1 and Arf2 GTPases are activated by three distinct GEFs operating sequentially as Golgi cisternae mature. Gea1 functions at early Golgi cisternae, whereas Gea2 associates with medial- and/or trans-Golgi compartments, and Sec7 localizes to the TGN (Bui et al., 2009; Highland and Fromme, 2021). The distribution of these GEFs can, therefore, serve to identify the maturation state of individual Golgi compartments. In static images obtained by confocal fluorescence microscopy, we observed that AP-3 in wild-type cells is more strongly colocalized with Gea2 than with Sec7. However, AP-3 shifts away from Gea2 toward Sec7 when AP-3 vesicle budding is impaired. These results suggest that AP-3 recruitment begins at medial- and/or late-Golgi compartments and continues as these compartments mature into the TGN. This inference is in line with time-lapse imaging studies tracking the arrival and departure of different adaptors relative to Sec7, which established an order of vesicle budding during the maturation of late Golgi compartments: AP-3 is the first adaptor recruited in this sequence and peaks in abundance as Sec7 arrives, whereas GGA recruitment coincides with that of Sec7 and is followed by AP-1 recruitment (Daboussi et al., 2012; Day et al., 2018; Tojima et al., 2019; Casler and Glick, 2020; Highland and Fromme, 2021).

Using super-resolution confocal fluorescence microscopy, we resolved the positional relationships between AP-3, GGA and AP-1 in more detail. In wild-type cells, Gga2 puncta were frequently seen in a radial pattern that was occasionally infiltrated by AP-3 puncta. In contrast, AP-3 budding-deficiency caused Gga2 and AP-3 puncta to extensively commingle but remain distinct from one another, alternating in a donut-shaped pattern. The circular distribution of Gga2 and AP-3 puncta correlates with our EM studies, which showed accumulation of AP-3 large subunits on rounded Golgi by immunolabeling. At the circular structures imaged by super-resolution confocal fluorescence microscopy, the alternating pattern of Gga2 and AP-3 puncta is consistent with these Golgi adaptors localizing to discrete membrane microdomains. This partitioning might explain why GGA sorting activity remains unperturbed in AP-3-budding-deficient cells despite the neighboring accumulation of AP-3. In wild-type cells, AP-3 and AP-1 puncta rarely overlapped with one another but, as with GGA, AP-1 colocalization with AP-3 doubled in AP-3-budding-defective cells. Thus, unbudded AP-3 remains associated with Golgi compartments as late as AP-1 recruitment, which peaks downstream of GGA recruitment during Golgi maturation (Daboussi et al., 2012; Day et al., 2018; Tojima et al., 2019; Highland and Fromme, 2021).

The hinge truncations that disable AP-3 budding also caused a shift in the localization patterns of Gea2 and Sec7. Each of these GEFs exhibit punctate or tubular distributions in wild-type cells, but both adopted a more rounded pattern of fluorescence in cells bearing AP-3 hinge truncations, much like we observed for AP-3 itself under these conditions. Again, this shape change correlated with the accumulation of round Golgi compartments in truncation-mutant cells, as observed by electron tomography, which was also seen in *apl5*Δ and *apl6*Δ deletion-mutant cells. Why Golgi structure might be impacted by AP-3 dysfunction has not been determined, although it seems likely to result from the continued presence of proteins and lipids that would otherwise have been removed from the Golgi by AP-3.

In conclusion, our study revealed that, despite lacking the ear domains characteristic of AP-1 and AP-2 complexes, the unstructured hinges of the δ (Apl5) and β3 (Apl6) subunits are essential for AP-3 function in yeast. Truncation of these hinges disrupted AP-3 vesicle budding but did not impair AP-3 complex recruitment to the Golgi, indicating a role for the intrinsically disordered hinges in vesicle formation and/or fission. Further investigation into the biophysical properties and potential interactors of AP-3 hinge regions and the interplay between AP-3 and other Golgi-associated proteins will be crucial to understanding the mechanism of AP-3-mediated cargo sorting and its effects on Golgi maturation.

## MATERIALS AND METHODS

### Computational modeling

Ribbon structures of the four adaptor protein subunits in each of the AP-1, AP-2 or AP-3 complexes in *S. cerevisiae* or in humans were generated by the predictive algorithm AlphaFold2 (Jumper et al., 2021). Molecular graphics and analyses of these structures were performed with UCSF ChimeraX (Meng et al., 2023), developed by the Resource for Biocomputing, Visualization, and Informatics at the University of California, San Francisco, with support from National Institutes of Health grant R01-GM129325 and the Office of Cyber Infrastructure and Computational Biology, National Institute of Allergy and Infectious Diseases. Intrinsically disordered regions in the Apl5 and Apl6 protein sequences were identified using the Metapredict algorithm (Emenecker et al., 2021).

### Construction of yeast strains and DNA plasmids

Standard techniques were used for the growth and genetic manipulation of *S. cerevisiae* strains (Table S1) and for the construction of plasmids (Table S2). Yeast strains created for this study were constructed by one-step PCR-based integration (Longtine et al., 1998), with the exception of strains expressing C-terminal GFP-tagged Apl5 or Apl6, which were created by chromosomal integration of linearized plasmids YIplac211-APL5-iGFPx6 or YIplac211-APL6-msGFPx3 (Day et al., 2018). Strains expressing the AP-3 synthetic cargo, GNSS, were also created by chromosomal integration of plasmid pLC1514 linearized using NotI-SnaBI, which integrates at the *SUC2* locus. All yeast strains in this study were authenticated by PCR analysis of genomic DNA. The wild-type *APL5* or *APL6* gene was cloned into the pRS416 yeast shuttle vector (Sikorski and Hieter, 1989) using the Gibson cloning method. The cloned *APL5* gene included 1000 bp of promoter sequence and 312 bp of terminator sequence. The cloned *APL6* gene included 571 bp of promoter sequence and 779 bp of terminator sequence. pRS416 plasmids encoding the series of truncation-mutant *apl5* or *apl6* alleles were created by introducing nonsense mutations in the corresponding wild-type gene using QuikChange mutagenesis (Agilent Technologies, Santa Clara, CA, USA). Bacterial expression plasmids encoding GST fused to hinge regions of different AP large subunits were constructed by PCR amplification of each region, digestion of each PCR product with NcoI and BamHI, and ligation of each digested product with NcoI- and BamHI-digested pGST-Parallel (Sheffield et al., 1999). All yeast and DNA reagents are available upon request.

### Yeast cell growth assays

The effects of adaptor subunit gene deletions on the growth of yeast strains expressing the wild-type *CHC1* gene or the temperature-sensitive *chc1-521* allele were performed as described previously (Yeung et al., 1999). Yeast cultures were grown overnight to saturation and adjusted the next day to the same density (1 OD_600_/ml), then serially diluted 10-fold, and cells (5 μl) were spotted onto growth medium. Each plate was incubated at 24°C, 30°C or 37°C for 2 days before imaging.

### Protein purification and binding assays

*Escherichia coli* strain BL21 (DE3) was transformed with different pGST-Parallel vectors encoding GST–hinge regions. Protein expression was induced by the addition of isopropyl β-D-1-thiogalactopyranoside to a final concentration of 200 μM. Cells were pelleted and resuspended in 40 ml of PBS buffer [phosphate buffered saline (pH 7.4), 1 mM DTT, 1 mM PMSF, 1 μg/ml leupeptin, 1 μg/ml pepstatin]. Approximately 700 µl of the cell suspension was supplemented with 0.5% Triton X-100 and 10 μg DNase I, then lysed by sonication, and centrifuged at 20,000 ***g*** for 20 min to produce clarified bacterial lysate. Then, 500 μl of the lysate was added to 50 μl Glutathione Sepharose 4B (Amersham Biosciences, Piscataway, NJ, USA), rotated overnight at 4°C, and washed thrice with 500 μl PBS, thrice with 500 μl PBS containing 350 mM NaCl, and thrice with 500 μl yeast lysis buffer {20 mM HEPES pH 6.8, 0.2 M sorbitol, 2 mM EDTA, 50 mM potassium acetate, 1 μg/ml aprotinin, 1 μg/ml leupeptin, 1 μg/ml pepstatin, 1 μg/ml Pefabloc-SC [4-(2-Aminoethyl)benzenesulfonyl fluoride hydrochloride], 1 mM PMSF}. Washed GST resin was then added to 500 μl (150 OD_600_ units) of yeast extract, which was produced by supplementing clarified yeast cell lysate prepared from 1 l cultures of yeast cells. To prepare yeast cell lysate, yeast cells were pelleted by centrifugation, converted to spheroplasts, then gently sedimented at 1000 ***g*** for 2 min. The pellet was resuspended in yeast lysis buffer and homogenized by 25 strokes in a 15-ml tissue grinder/Dounce homogenizer to produce clarified yeast cell lysate, in which unlysed cells were removed by sedimentation at 1000 ***g*** for 5 min. GST affinity resin was incubated with yeast lysate for 1 h at 4°C, then washed thrice with ice-cold yeast lysis buffer, before incubating with 50 μl glutathione elution buffer (50 mM Tris-Cl pH 7.9, 20 mM reduced glutathione, 600 mM NaCl, 1% Triton X-100) for 10 min on ice. Two elutions were pooled and resolved by SDS polyacrylamide gel electrophoresis. Gels were stained with Coomassie Brilliant Blue G, or transferred to nitrocellulose and imaged by western blotting using rabbit antiserum against yeast clathrin heavy chain (Lemmon et al., 1988).

### Invertase activity assays

The activity of extracellular invertase secreted by cells growing on solid support was assessed by spotting cells as described above to agar medium in which fructose was substituted in place of glucose as the carbon source. The cells were incubated at 26°C for 2 days before being overlaid with top agar containing a chromogenic solution [125 mM sucrose (ultrapure), 166 mM sodium acetate (pH 5.2), 0.666 mM *N*-ethylmaleimide, 0.017 mg/ml horseradish peroxidase, 15 units/ml glucose oxidase, 1 mg/ml *o*-dianisidine, 3% agar (w/v)]. Secreted invertase activity is observed as a color change from white to reddish-brown within ∼15 min (Darsow et al., 2000). Quantitation of secreted invertase activity was assayed by centrifuging 0.4 OD_600_ units of cells grown in culture to mid-logarithmic density and washing the cells twice in 100 mM sodium acetate (pH 5.2), before resuspending them in 400 µl of 100 mM sodium acetate (pH 5.2). These samples were then split into two groups of 190 μL each, one to measure extracellular invertase activity and the other to measure total invertase activity, using the liquid invertase assay described in Darsow et al. (2000). Quantitative measurements of the amounts of secreted invertase activity were performed in triplicate in at least three independent experiments.

### Fluorescence microscopy

Liquid yeast cultures were grown to mid-log phase before staining the vacuole lumen using CMAC blue stain (Thermo Fisher Scientific) for 20 min in yeast extract peptone dextrose and then rinsed prior to imaging. For conventional spinning disk confocal fluorescence microscopy, live yeast cells were observed at room temperature (RT) with an inverted fluorescence microscope (Ti2 2E PSF; Nikon) equipped with a Yokogawa CSU-X1 spinning disk confocal system and a 100× (1.45 NA) oil objective (Plan Apo λ; Nikon). Images taken with an Andor iXON Ultra 512×512 EMCCD camera were acquired with Micromanager version 2.0 software and analyzed with ImageJ software (National Institutes of Health). For super-resolution spinning disk confocal microscopy, live cells were imaged at RT with an inverted fluorescence scope (Ti2 2E PSF; Nikon) equipped with a Yokagawa CSU-W1SoRa (super-resolution through optical reassignment) spinning disc confocal system and a 60× (1.42 NA) oil objective (Plan Apo λD; Nikon). Images were acquired in NikonElements using a Hamamatsu ORCA-FUSION-BT C15540 camera and magnified using the intermediate 2.8× magnifier. These images were then deconvolved using NikonElements 3D deconvolution package and analyzed further using ImageJ software. Colocalization frequency in super-resolution images was performed by maximally projecting three *z*-stacks at 0.5 µm spacing. The projected images were normalized to a set of wild-type images for fluorescence intensity. ImageJ thresholding was used to generate masks of cell boundaries and outline the puncta of proteins of interest. These masks were used to measure pixel overlap of proteins of interest, puncta abundance and puncta size of each channel. Colocalization events were quantified relative to each channel and were filtered for >5% pixel overlap before quantification. At least 200 cells were sampled for each experimental condition. Each condition was repeated on separate days for at least three experimental replicates, and the standard error of the mean was calculated. Statistical significance was calculated in GraphPad Prism software using paired and unpaired two-tailed Student's *t*-test, as appropriate.

For VAEF/HiLo microscopy, yeast samples were seeded onto agarose pads as described previously (Rines et al., 2011). Spinning disk and high-speed images were acquired using a Nikon Ti2 frame with a Mad City piezoelectric *z*-stage and a 100×1.45 NA Plan Apochromat objective. High-speed imaging was done with a Toptica iChrome MLE laser combiner and launch, 488 and 561 nm diode lasers (Coherent), a Gataca Systems iLas2 laser aiming module operated in 360° HiLo mode, a Cairn OpoSplit II Bypass emission splitting unit, a custom filter and dichroic mirror set (Chroma, Brattleboro, VT, USA) and a Photometrics Prime 95B sCMOS camera operated at frame rates of 20–80 Hz. Pixel spacing at the sample focal plane, with a 1.5× tube lens, was 73 nm. Images were deconvolved using NikonElements software to reduce noise and analyzed using ImageJ. Particle tracking for fluorescent proteins was performed using the ImageJ TrackMate plugin (Ershov et al., 2022). Particle tracks were filtered using a threshold of at least 20 persistent frames (1 s) before quantification. Track maps and data were exported to XML files and then analyzed in MATLAB using the @msdanalyzer tool to generate the mean squared displacement (MSD) (graphs presented in Fig. 4C,D) (Tarantino et al., 2014). Vesicle tracks from at least 30 cells per experimental condition were used to generate the MSD graphs and confidence intervals. The data were acquired in three independent experiments over several days.

### Electron microscopy

Liquid cultures of yeast cells were harvested at mid-log phase, vacuum-filtered on 0.45-μm Millipore paper, loaded into 0.5-mm aluminum hats, and high-pressure frozen with a high-pressure freezer (Wohlwend, Switzerland). Cells were freeze-substituted in an Automated Freeze-Substitution machine (AFS, Leica Vienna, Austria) at −90°C in an *en bloc* preparation of 0.1% uranyl acetate and 0.25% glutaraldehyde in anhydrous acetone. Samples were then washed in pure anhydrous acetone, embedded in Lowicryl HM20 resin (Polysciences, Warrington, PA, USA), and polymerized using ultraviolet light at −60°C before warming slowly over 4 days to RT. The sample blocks were then stored at −20°C. These methods preserve membrane and protein structure and provide consistent *en bloc* staining for immuno-EM membrane identification (Giddings, 2003).

A Leica UC6 Ultra-Microtome was used to cut and place serial sections on Formvar-coated rhodium-plated copper slot grids (Electron Microscopy Sciences). 80- to 90-nm serial sections were cut for transmission EM and immuno-EM experiments, and 200-nm thick serial sections were cut for dual-axis tomography. For immunolabeling experiments, grids were exposed to sequential 50 μl droplets. Nonspecific antibody binding was blocked by incubation with 1% PBS containing 1% dry milk (blocking solution) for 20 min at RT, then exposed to primary antibodies overnight at 4°C (1:200 anti-GFP, 11814460001, Roche) in blocking solution, washed at RT in 1% PBS with three sequential 50 μl drops, labeled with a secondary anti-rabbit or anti-mouse gold (depending on the primary antibody used) at RT for 1 h (1:100 goat anti-rabbit IgG, A6154-1ML, Sigma-Aldrich, Electron Microscopy Sciences), washed in 1% PBS with three sequential 50 μl drops, and finally washed in distilled water with two sequential 50 μl drops.

Immuno-EM sections were imaged with a FEI Tecnai T12 Spirit electron microscope equipped with a 120 kV LaB6 filament and AMT (2k×2k) CCD. Images from a hundred randomly oriented cells per strain were used to quality-control freezing, embedding and staining. Thick sections were labeled with fiduciary 15-nm colloidal gold (British Biocell International) on both sides and tilt imaged with a Tecnai 30 (f-30, 300 kV; FEI-Company, Eindhoven, the Netherlands), with dual-tilt series images collected from +60° to −60° with 1.5° increments using a Gatan US4000 4k×4k CCD camera (Abingdon, UK). The tilt series were imaged primarily at 19,000× magnification and repeated with a 90° rotation for dual-axis tomography (Mastronarde, 1997). Tomograms were built and modeled using the IMOD software package (Kremer et al., 1996) using an iMac (Apple). Golgi, vacuoles and cytosolic vesicle models from tomograms were manually assigned from the outer leaflet every 5 nm. Videos were made using IMOD and QuickTime Pro (Apple). Data were analyzed and graphed using Prism 9 (GraphPad).

## Supplementary Material



10.1242/joces.262234_sup1 Supplementary information
