# Supplementary material for: Disordered hinge regions of the AP-3 adaptor complex promote vesicle budding from the late Golgi in yeast
Source: J Cell Sci. 2024 Nov 8;137(21):jcs262234. doi: 10.1242/jcs.262234 (PMC11574352; doi:10.1242/jcs.262234)
Supplement: Supplementary information [file joces-137-262234-s1.pdf]

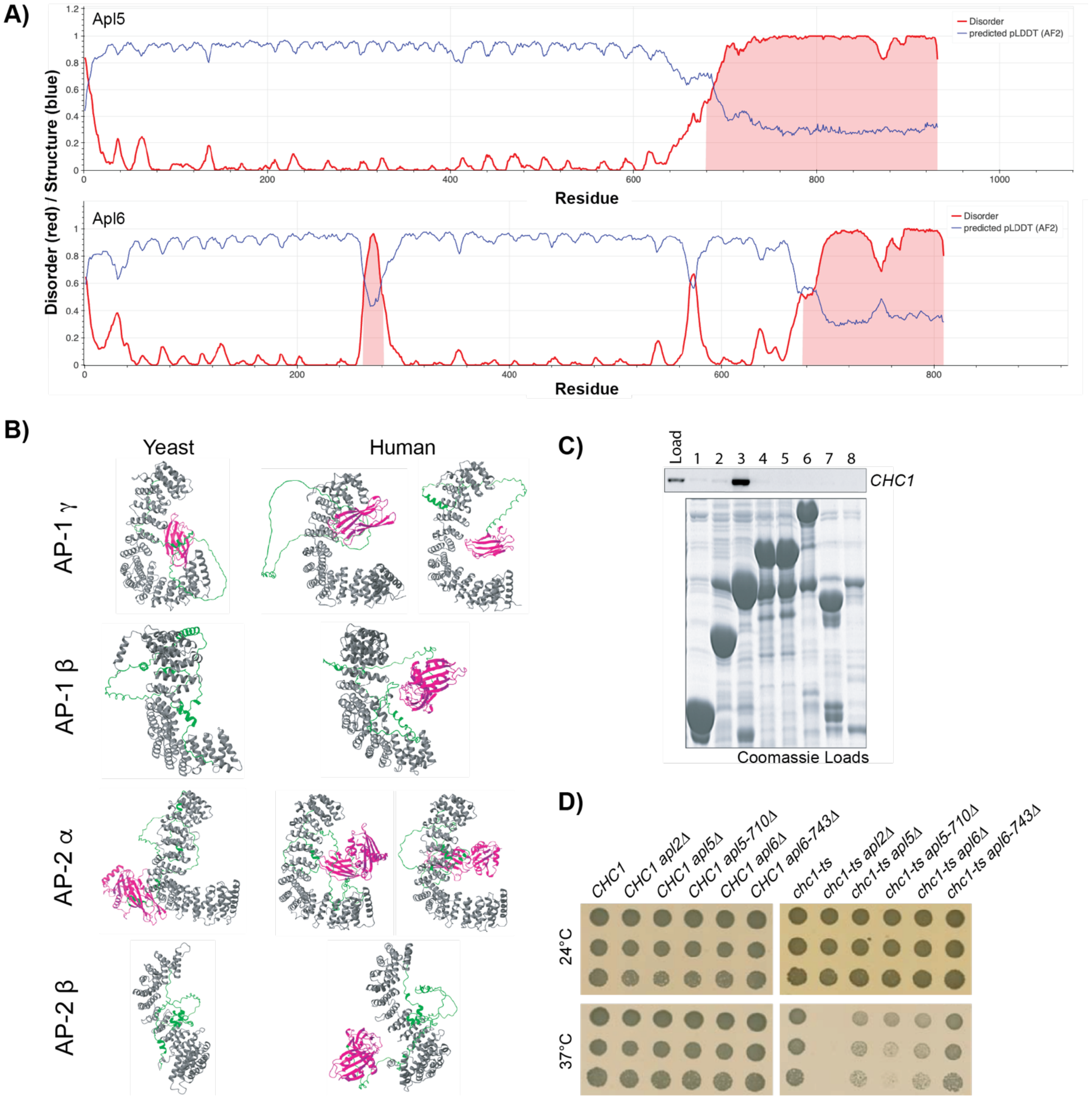

Fig. S1. Ear domains are absent from yeast AP-3 complexes in computational models. A) Metapredict data of Apl5 and Apl6 defining predicted ordered (blue) and disordered regions (red). B) AlphaFold2 predicted structures of large subunits of yeast and human AP-1 and AP-2. Grey coloring indicates the trunk region of each subunit, green shows the hinge regions, and magenta shows the structured ear domain, if predicted. C) Clathrin in yeast lysates binds purified Apl4-hinge (yeast AP-1) fused to GST, but not Apl5-hinge or Apl6-hinge fused to GST. Upper panel shows anti-clathrin heavy chain immunoblot of samples pulled down by glutathione resin after incubation of yeast lysate with each of the GST fusions, as follows: GST only (lane 1), GST-Apl4 amino acids 717-832 (lane 2), GST Apl4 amino acids 662-832 (lane 3), GST-Apl5 amino acids 711-932 (lane 4), GST-Apl5 amino acids 711-932, with the clathrin binding motif-like sequence mutated (lane 5), GST-Apl5 amino acids 615- 932 (lane 6), GST-Apl6 amino acids 673-809 (lane 7), and GST-Apl6 amino acids 625-809 (lane 8). Lower panel, coomassie-stained gel showing the amount of each GST fusion protein used in each pulldown. D) Yeast cell growth assay of the effects of temperature-sensitive clathrin heavy chain mutants when paired with AP complex subunit mutations at 24˚C and 37˚C.

Journal of Cell Science • Supplementary information

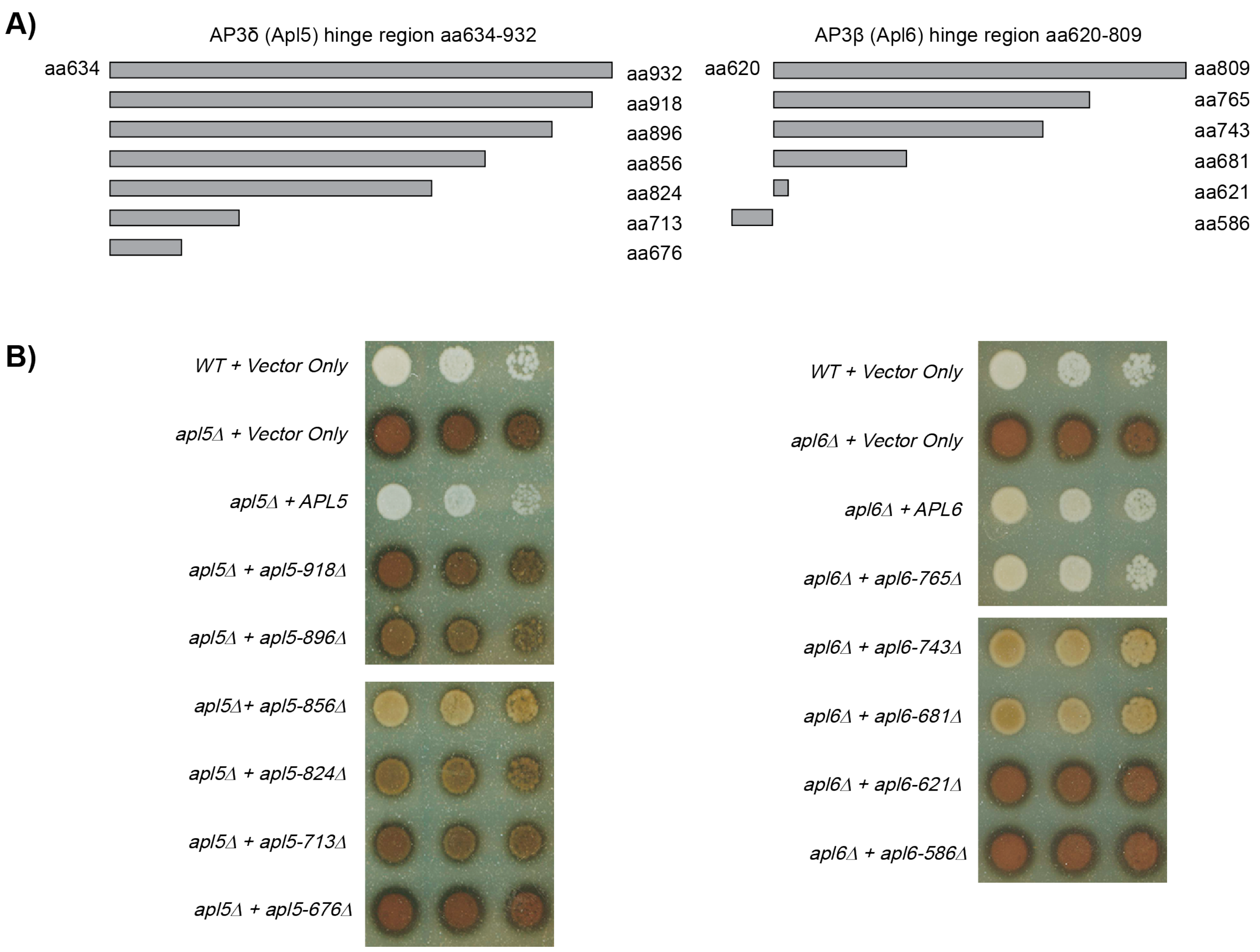

Fig. S2. AP-3 trafficking in yeast requires the hinge of each AP-3 large subunit. A) Diagrams depict the amount of C-terminal hinge region (grey boxes) in each of the *apl5* and *apl6* truncation-mutant alleles that were analyzed in (B) by overlay reaction to detect GNSS missorting to the plasma membrane. Full-length Apl5 is 932 amino acids; full-length Apl6 is 809 amino acids. B) Overlay reaction (see Materials and Methods) of agar medium spotted with a dilution series of wild-type cells versus *apl5*Δ or *apl6*Δ cells transformed with low-copy plasmids encoding the indicated *apl5* or *apl6* truncation-mutant alleles. Each overlay reaction was conducted for 30 minutes before imaging the plate. Cellular concentrations for each strain were 1.0, 0.1, and 0.01 OD_{600} units/mL (left to right). The relatively weak GNSS missorting phenotype caused by the longer *apl5-856*Δ truncation in comparison to shorter truncations (*apl5-918*Δ and *apl5-896*Δ) might signify a structural distortion that impacts Apl5 function when intermediate lengths of the hinge region are present, though such a possibility would require further analysis. Note that the relative amount of GNSS by *apl5-713*Δ cells, which is qualitatively detected by overlay in Supplemental Figure 2B, is consistent with the fraction (80%) of GNSS missorted by *apl5- 710*Δ cells, which was measured quantitatively in Figure 2B. The *apl5-710*Δ allele was used for all other experiments in this study because our prior results showed that the region of the hinge deleted by the *apl5-710*Δ truncation (amino acids 711-932) directly binds Vps41, a subunit of the HOPS complex that tethers AP-3-coated vesicles to the vacuole (Angers and Merz, 2009).

Journal of Cell Science • Supplementary information

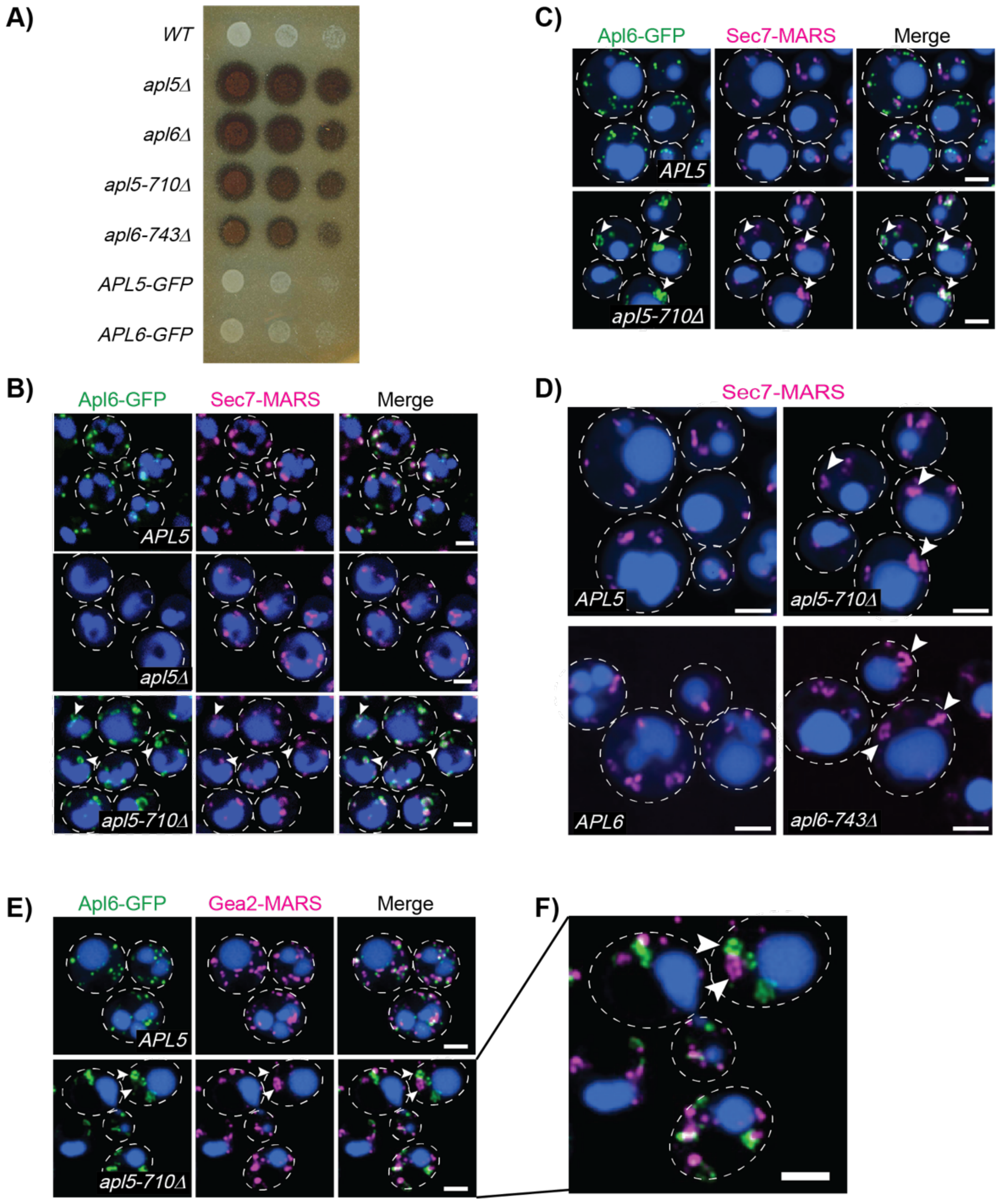

Fig. S3. Truncation of either hinge region causes AP-3 accumulation at the Golgi. All scale bars are 2 µm. A) GNSS overlay of strains expressing Apl5 or Apl6 truncation mutants or expressing GFP-tagged Apl5 or Apl6. Overlay reaction was allowed to continue for 30 minutes before imaging the plate. Cellular concentration for each strain was 1, 0.1, and 0.001 OD_{600} units/mL (left to right). B) Images showing AP-3 localization relative to Sec7. AP-3 subunits tagged with GFP were expressed at their endogenous loci. Sec7-MARS was expressed from the pRS415 plasmid. The vacuole lumen was stained with CMAC Blue. C) Super-resolution confocal fluorescence microscopy showing distribution of AP-3 relative to Sec7. Arrowheads indicate rounded structures for both Sec7 and AP-3. D) Zoomed in image of Sec7 showing abnormal structure in AP-3 hinge truncation mutants. Arrowheads indicate round structures. E) Super-resolution confocal fluorescence microscopy showing distribution of Apl6-GFP relative to Gea2-Mars. Arrowheads indicate rounded structures for both Gea2-Mars and Apl6-GFP. F) Zoomed in image of Gea2 and AP-3 round structures in AP-3 truncation mutants.

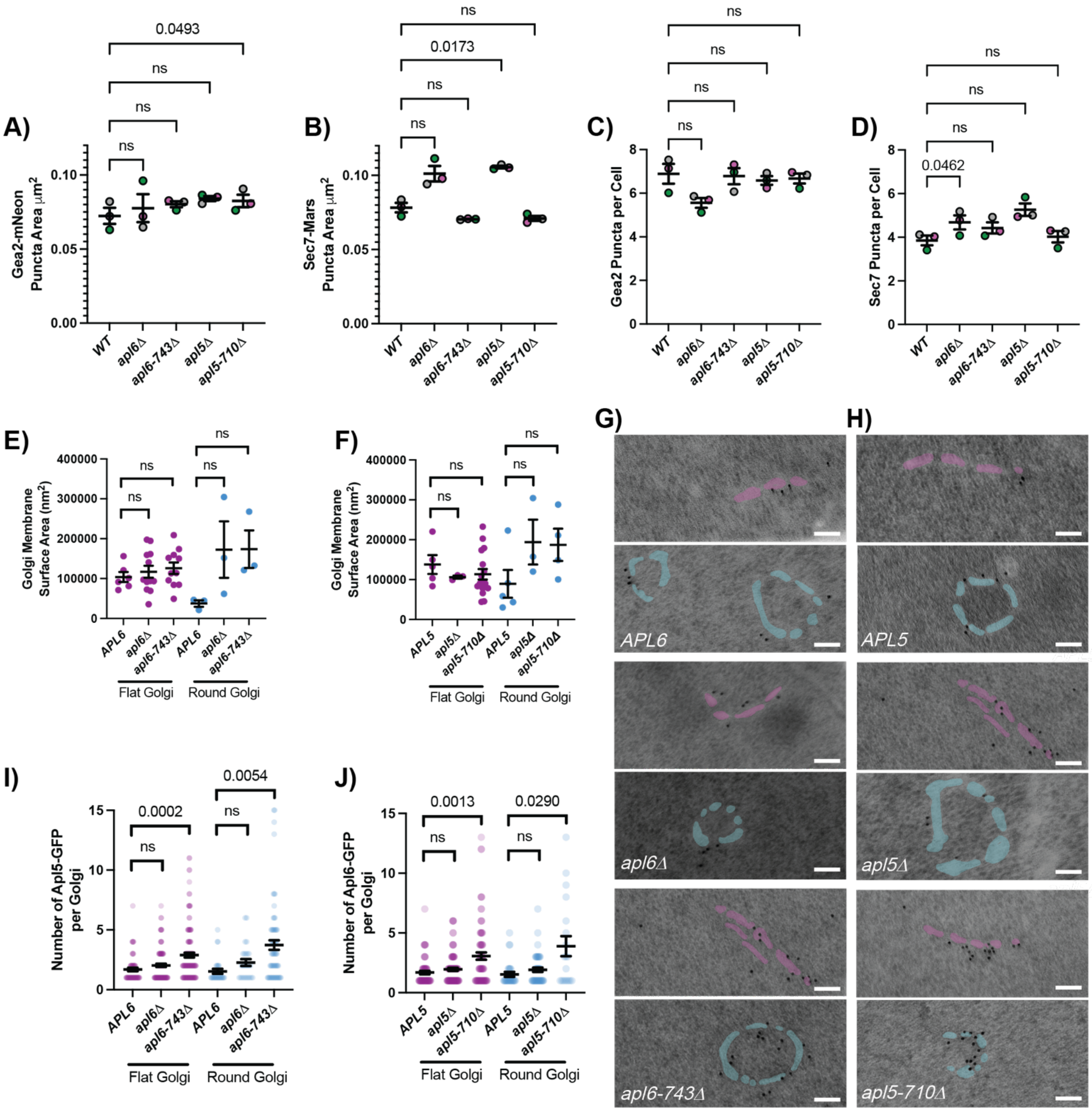

Fig. S4. AP-3 dysfunction alters Golgi morphology. A-B) Quantitation of average puncta size by area in square microns of Gea2-mNeon (A) and Sec7-Mars (B). Each point represents the averages of at least 200 cells. Error bars show SEM. P-values were determined by Dunett's test. C-D) Quantitation of average count of puncta per cell of Gea2-mNeon (C) and Sec7-Mars (D). Each point represents the averages of at least 200 cells. Error bars show SEM. P-values were determined by Dunett's test. E-F) Quantitation of the average membrane surface areas of flat and round Golgi in *apl6* mutant cells (E) and *apl5* mutant cells (F). Error bars show SEM. Magenta bars represent flat Golgi data; teal bars represent round Golgi data. P-values were determined using Dunnett's test. G-H) Immuno-gold labeling of Apl5-GFP (G) and Apl6-GFP (H) in wild-type and mutant AP-3 strains. Upper panels show flat Golgi in magenta while lower panels show round Golgi in cyan. Scale bars indicate 100 nm. I-J) Quantitation of the number of gold particles observed by immuno-EM using anti-GFP antibodies. Error bars show SEM. Magenta bars represent flat Golgi data, teal bars represent round Golgi data. P-values were determined using Dunnett's test.

Journal of Cell Science • Supplementary information

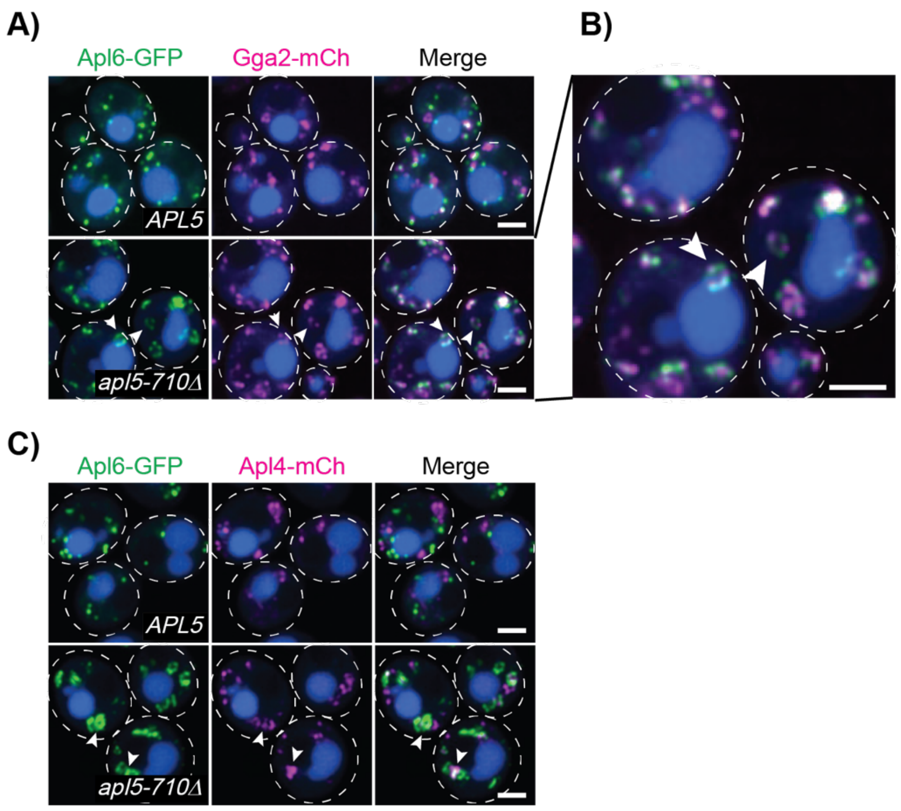

Fig. S5. Budding deficiency causes AP-3 to accumulate onto late Golgi adaptors. All scale bars are 2 μm. A) Super-resolution confocal fluorescence microscopy showing the distribution of AP-3 relative to Gga2. The vacuole lumen was stained with CMAC Blue and cell outlines are shown with white dotted lines. Arrowheads indicate rounded structures for both Gga2 and AP-3. B) Zoomed in image of AP-3 and GGA commingling donuts. C) Super-resolution confocal fluorescence microscopy showing distribution of AP-3 relative to AP-1. AP-3 subunits tagged with GFP and Apl4 tagged with mCherry were expressed at their endogenous loci. The vacuole lumen was stained with CMAC Blue and cell outlines are shown with white dotted lines. Arrowheads indicate rounded structures for both AP-1 and AP-3.

Journal of Cell Science • Supplementary information

Table S1. Yeast strains used in this study

Strain Name	Genotype	Reference
BY4742	*MATα his3Δ1 leu2Δ0 lys2Δ0 ura3Δ0*	ATCC
GPY1100	*MATalpha leu2-3,112 ura3-52 his4-519 trpl canl*	Payne and Schekman, 1989
GOY844	GPY1100*; apl2Δ::KANMX6*	This Study
GOY822	GPY1100*; apl5Δ::KANMX6*	This Study
GOY863	GPY1100*; apl5-710Δ::KANMX6*	This Study
GOY891	GPY1100*; apl6Δ::KANMX6*	This Study
GOY861	GPY1100*; apl6-743Δ::KanMX6*	This Study
GPY418	*MATalpha leu2-3,112 ura3-52 his4-519 trpl canl; chc1-521*	Tan et al., 1993
GOY845	GPY418*; apl2Δ::KANMX6*	This Study
GOY823	GPY418*; apl5Δ::KANMX6*	This Study
GOY864	GPY418*; apl5-710Δ::KANMX6*	This Study
GOY894	GPY418*; apl6Δ::KANMX6*	This Study
GOY862	GPY418*; apl6-743Δ::KANMX6*	This Study
JTY01	BY4741*; suc2ΔGNSS::NATMX*	This Study
GOY696	BY4741*; suc2ΔGNSS::NATMX; apl5Δ::HIS3MX6*	This Study
GOY697	BY4741*; suc2ΔGNSS::NATMX; apl5-710Δ::HIS3MX6*	This Study
LDY49	BY4741*; suc2ΔGNSS::NATMX; apl6Δ::HIS3MX6*	This Study
GOY741	BY4741*; suc2ΔGNSS::NATMX; apl6-743Δ::HIS3MX6*	This Study
GOY788	BY4742*; vam3Δ::KANMX6; suc2Δ::HIS3MX6*	This Study
GOY760	SEY6210*; Apl5-iGFPx6::URA3*	This Study
GOY761	SEY6210*; Apl5-iGFPx6::URA3; apl6Δ::HIS3MX6*	This Study
GOY763	SEY6210*; Apl5-iGFPx6::URA3; apl6-743Δ::HIS3MX6*	This Study
GOY699	SEY6210*; Apl6-msGFPx3::URA3*	This Study
GOY702	SEY6210*; Apl6-msGFPx3::URA3; apl5Δ::HIS3MX6*	This Study
GOY703	SEY6210*; Apl6-msGFPx3::URA3; apl5-710Δ::HIS3MX6*	This Study

CFY2401	SEY6210a; *Gea2-3xMars::TRP1*	Highland and Fromme, 2021
MJLY129	SEY6210a; *Gea2-3xMars::TRP1; Apl5-iGFPx6::URA3*	This Study
MJLY122	SEY6210a; *Gea2-3xMars::TRP1; Apl5-iGFPx6::URA3; apl6-743Δ::HIS3MX6*	This Study
MJLY118	SEY6210a; *Gea2-3xMars::TRP1; Apl6-msGFPx3::URA3*	This Study
MJLY120	SEY6210a; *Gea2-3xMars::TRP1; Apl6-msGFPx3::URA3; apl5-710Δ::HIS3MX6*	This Study
CFY2378	SEY6210a; *Gea2-mNeon::HIS3; Sec7-Mars::TRP1*	Gustafson and Fromme, 2017
MJLY126	SEY6210a; *Gea2-mNeon::HIS3; Sec7-Mars::TRP1; apl5Δ::KANMX6*	This Study
MJLY107	SEY6210a; *Gea2-mNeon::HIS3; Sec7-Mars::TRP1; apl5-710Δ::KANMX6*	This Study
MJLY127	SEY6210a; *Gea2-mNeon::HIS3; Sec7-Mars::TRP1; apl6Δ::KANMX6*	This Study
MJLY108	SEY6210a; *Gea2-mNeon::HIS3; Sec7-Mars::TRP1; apl6-743Δ::KANMX6*	This Study
GOY875	SEY6210; *Apl5-iGFPx6::URA3; Gga2-mCherry::hphMX6*	This Study
GOY851	SEY6210; *Apl5-iGFPx6::URA3; Gga2-mCherry::hphMX6; apl6-743Δ::HIS3MX6*	This Study
GOY880	SEY6210; *Apl6-msGFPx3::URA3; Gga2-mCherry::hphMX6*	This Study
GOY881	SEY6210; *Apl6-msGFPx3::URA3; Gga2-mCherry::hphMX6; apl5-710Δ::HIS3MX6*	This Study
GOY819	SEY6210; *Apl5-iGFPx6::URA3; Apl4-mCherry::hphMX6*	This Study
GOY821	SEY6210; *Apl5-iGFPx6::URA3; Apl4-mCherry::hphMX6; apl6-743Δ::HIS3MX6*	This Study
GOY878	SEY6210; *Apl6-msGFPx3::URA3; Apl4-mCherry::hphMX6*	This Study
GOY879	SEY6210; *Apl6-msGFPx3::URA3; Apl4-mCherry::hphMX6; apl5-710Δ::HIS3MX6*	This Study
SEY6210	*MATα leu2-3,112 ura3-52 his3Δ200 trp1-Δ901 lys2-Δ801 suc2-Δ9*	Robinson et al., 1988
GOY4	SEY6210; *apl5Δ::HIS3*	Cowles et al., 1997

Journal of Cell Science • Supplementary information

apl6Δ	SEY6210; *apl6Δ::HIS3*	Cowles et al., 1997
GOY700	SEY6210; *apl5-710Δ::HIS3MX6*	This Study
GOY627	SEY6210; *apl6-743Δ::HIS3MX6*	This Study
GPY2151	SEY6210; *gga1Δ::HIS3*	Costaguta et al., 2001
GPY2149	SEY6210; *gga2Δ::HIS3*	Costaguta et al., 2001
GPY3431	SEY6210; *gga1Δ::HIS3; gga2Δ::HIS3*	Costaguta et al., 2006
AMY2460	SEY6210; *Apl5-ttx-GFP::TRP1; apl6Δ::KANMX4*	This Study

Table S2. Plasmids used in this study

Plasmid Name	Genotype	Reference
AMB501	*GST-APL4*$^{717\text{-}832}$	Angers and Merz, 2009
AMB508	*GST-APL4*$^{622\text{-}832}$	This Study
AMB436	*GST-APL5*$^{711\text{-}932}$	Angers and Merz, 2009
AMB499	*GST-APL5*$^{711\text{-}932\ cbm\Delta}$	This Study
AMB504	*GST-APL5*$^{615\text{-}932}$	This Study
AMB437	*GST-APL6*$^{673\text{-}809}$	Angers and Merz, 2009
AMB506	*GST-APL6*$^{625\text{-}809}$	This Study
pParallel-1-GST	*GST*	Sheffield et al., 1999
pLC1514	*GNSS; CEN URA3*	This Study
AMB1918	*APL5*$^{1\text{-}918}$ *in pRS416*	This Study
AMB1917	*APL5*$^{1\text{-}869}$ *in pRS416*	This Study
AMB1916	*APL5*$^{1\text{-}856}$ *in pRS416*	This Study
AMB1915	*APL5*$^{1\text{-}824}$ *in pRS416*	This Study
AMB1914	*APL5*$^{1\text{-}713}$ *in pRS416*	This Study
AMB1913	*APL5*$^{1\text{-}676}$ *in pRS416*	This Study
AMB1614	*APL5 in pRS416*	This Study
AMB1602	*APL6*$^{1\text{-}765}$ *in pRS416*	This Study
AMB1514	*APL6*$^{1\text{-}743}$ *in pRS416*	This Study
AMB1601	*APL6*$^{1\text{-}681}$ *in pRS416*	This Study
AMB1600	*APL6*$^{1\text{-}621}$ *in pRS416*	This Study
AMB1599	*APL6*$^{1\text{-}586}$ *in pRS416*	This Study
AMB743	*APL6 in pRS416*	This Study
pLT45	*mRFPmars-Sec7 in pRS415*	Thomas and Fromme, 2016
Ylplac211-Apl5-iGFPx6	*APL5-iGFPx6::URA3*	Day et al., 2018
Ylplac211-Apl6-msGFPx3	*APL6-msGFPx3::URA3*	Day et al., 2018
pFA6a-mChery-hphMX6	*mCherryx6::hphMX6*	Wang et al., 2014
pCYI50	*CPY-Inv; CEN URA3*	Johnson et al., 1987

Journal of Cell Science • Supplementary information

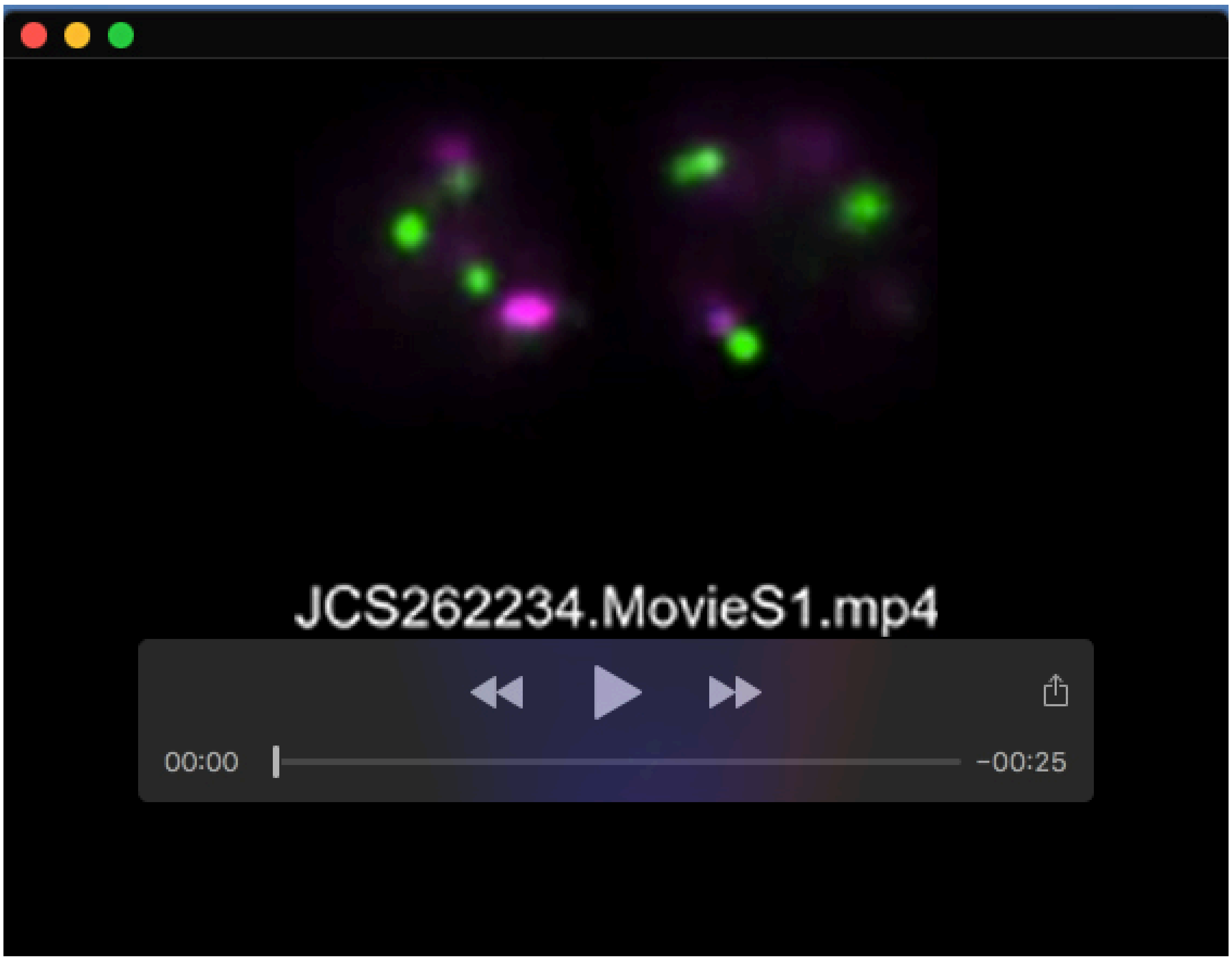

Movie 1. 30-second movie of wild-type *APL6* cells expressing Apl5-GFP and MARS-Sec7. Frames were taken at 50-ms intervals in a single plane.

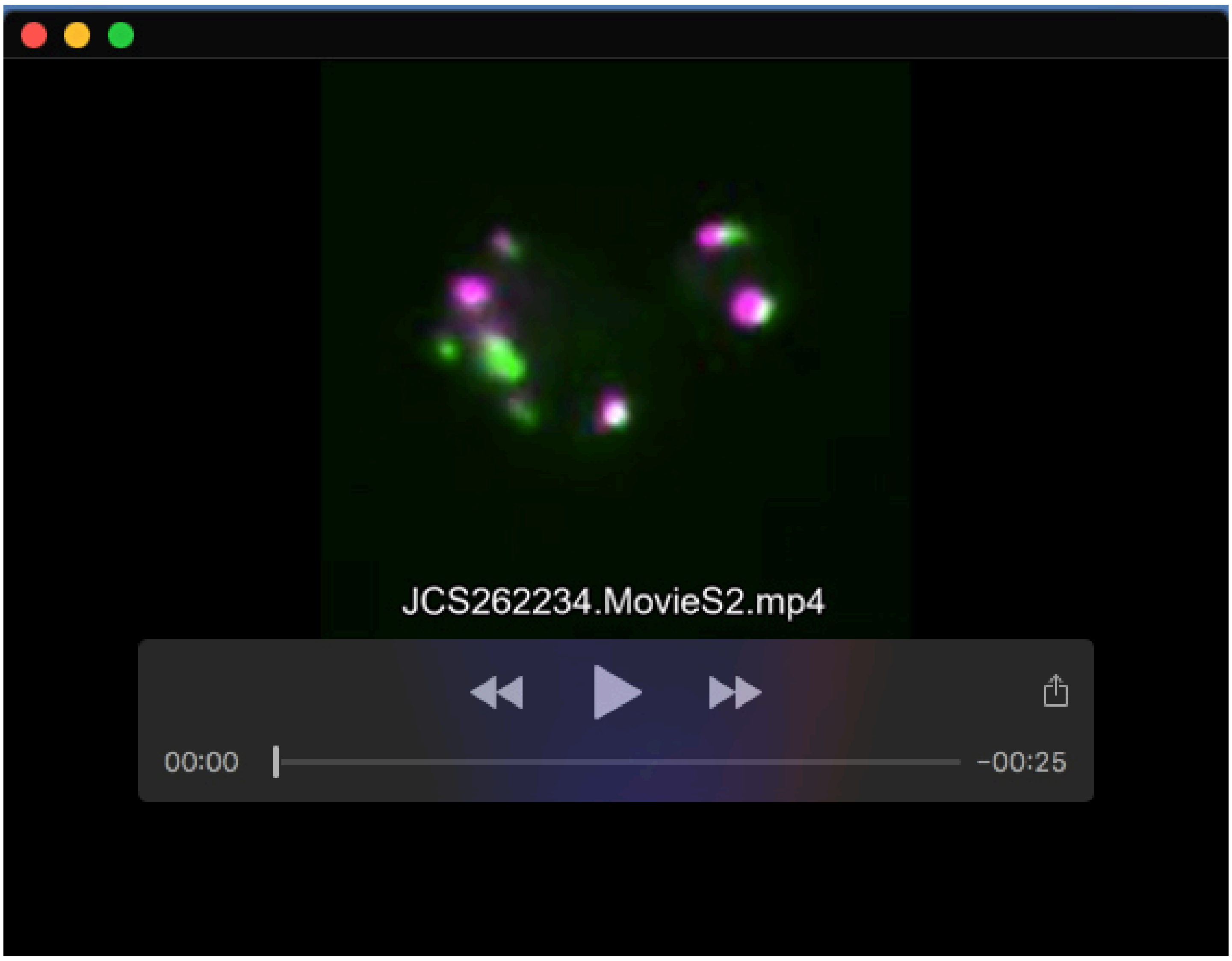

Movie 2. 30-second movie of *apl6-743*Δ cells expressing Apl5-GFP and MARS-Sec7. Frames were taken at 50-ms intervals in a single plane.

Journal of Cell Science • Supplementary information

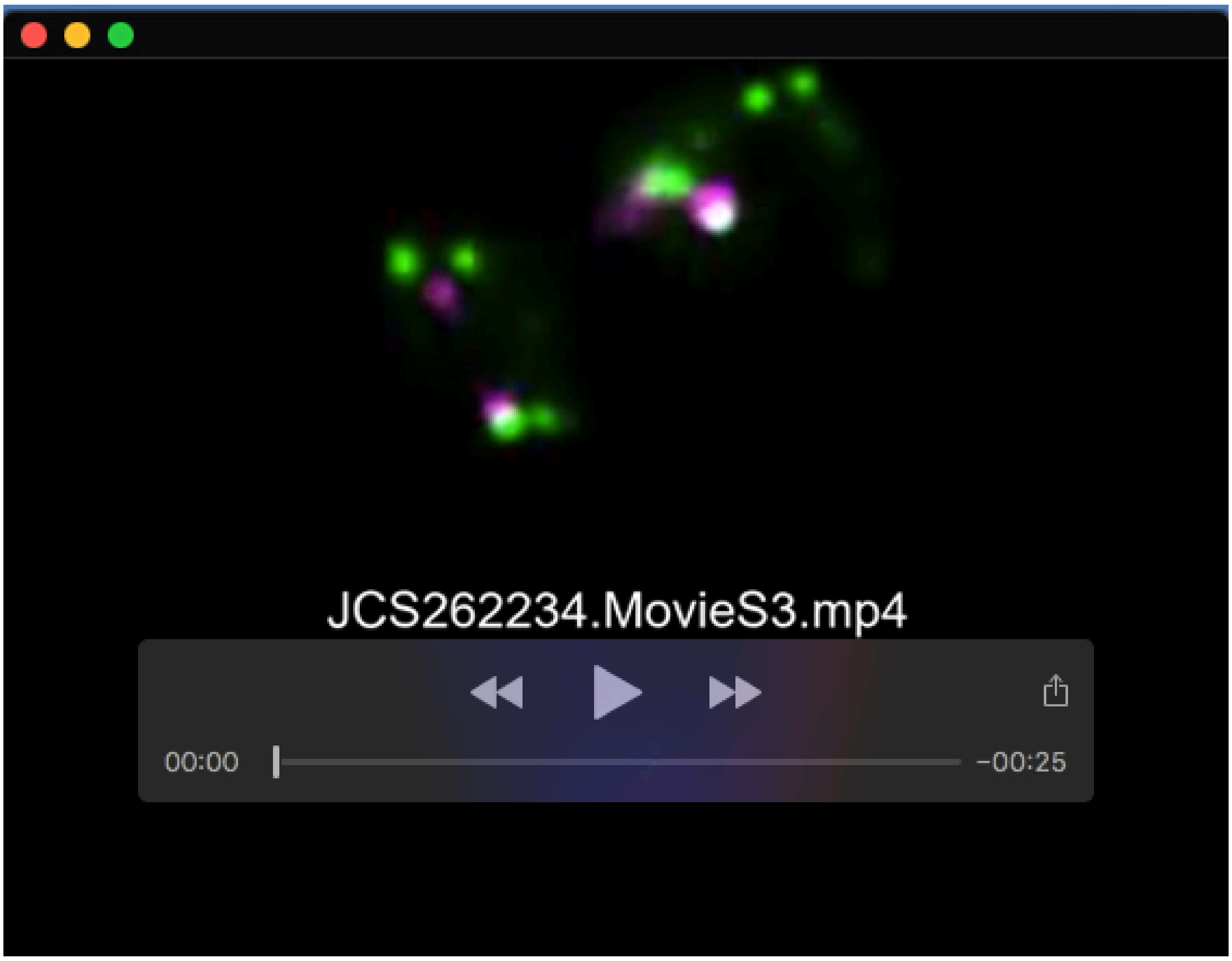

Movie 3. 30-second movie of wild-type *APL5* cells expressing Apl6-GFP and MARS-Sec7. Frames were taken at 50-ms intervals in a single plane.

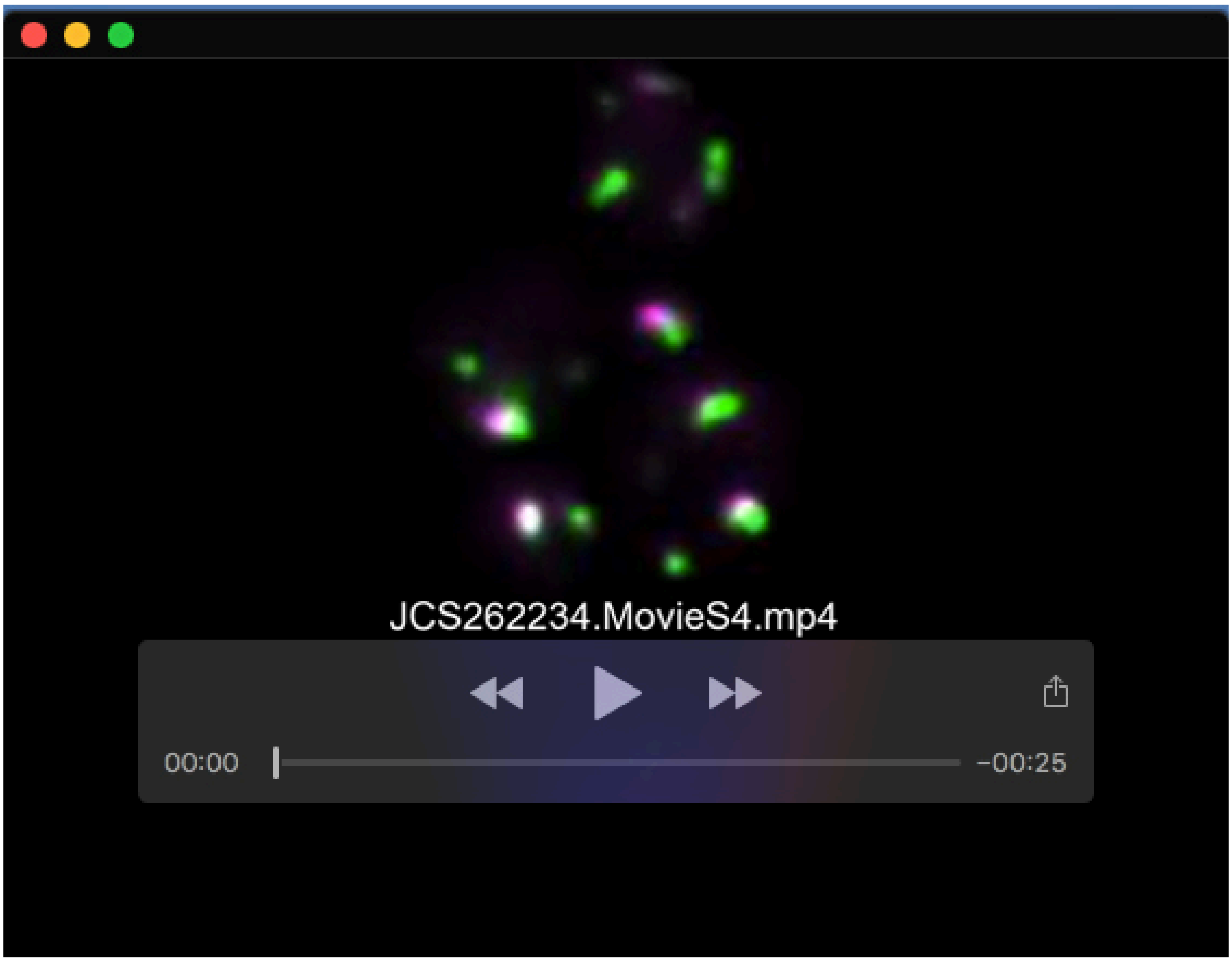

Movie 4. 30-second movie of wild-type *apl5-710*Δ cells expressing Apl6-GFP and MARS-Sec7. Frames were taken at 50-ms intervals in a single plane.

Journal of Cell Science • Supplementary information

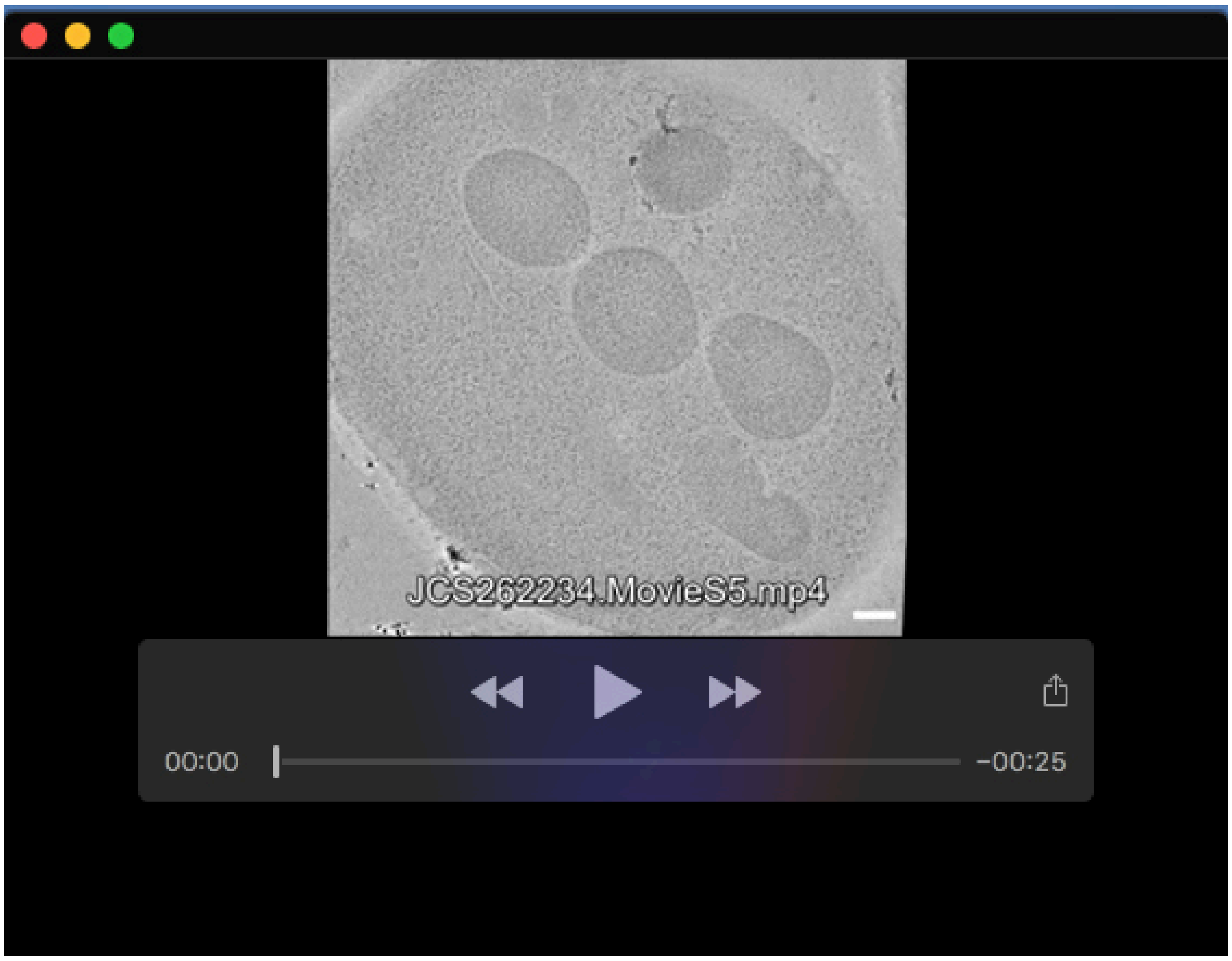

Movie 5. EM tomography data showing 3-D renderings of Golgi, vacuoles, and vesicles in *APL6* cells. Flat Golgi shown in magenta, round Golgi shown in cyan, curved Golgi shown in purple, vacuole membrane shown in red, and cytosolic vesicles shown in grey. Scale bars show 200 nm when zoomed out (beginning) and 100 nm when zoomed in (end).

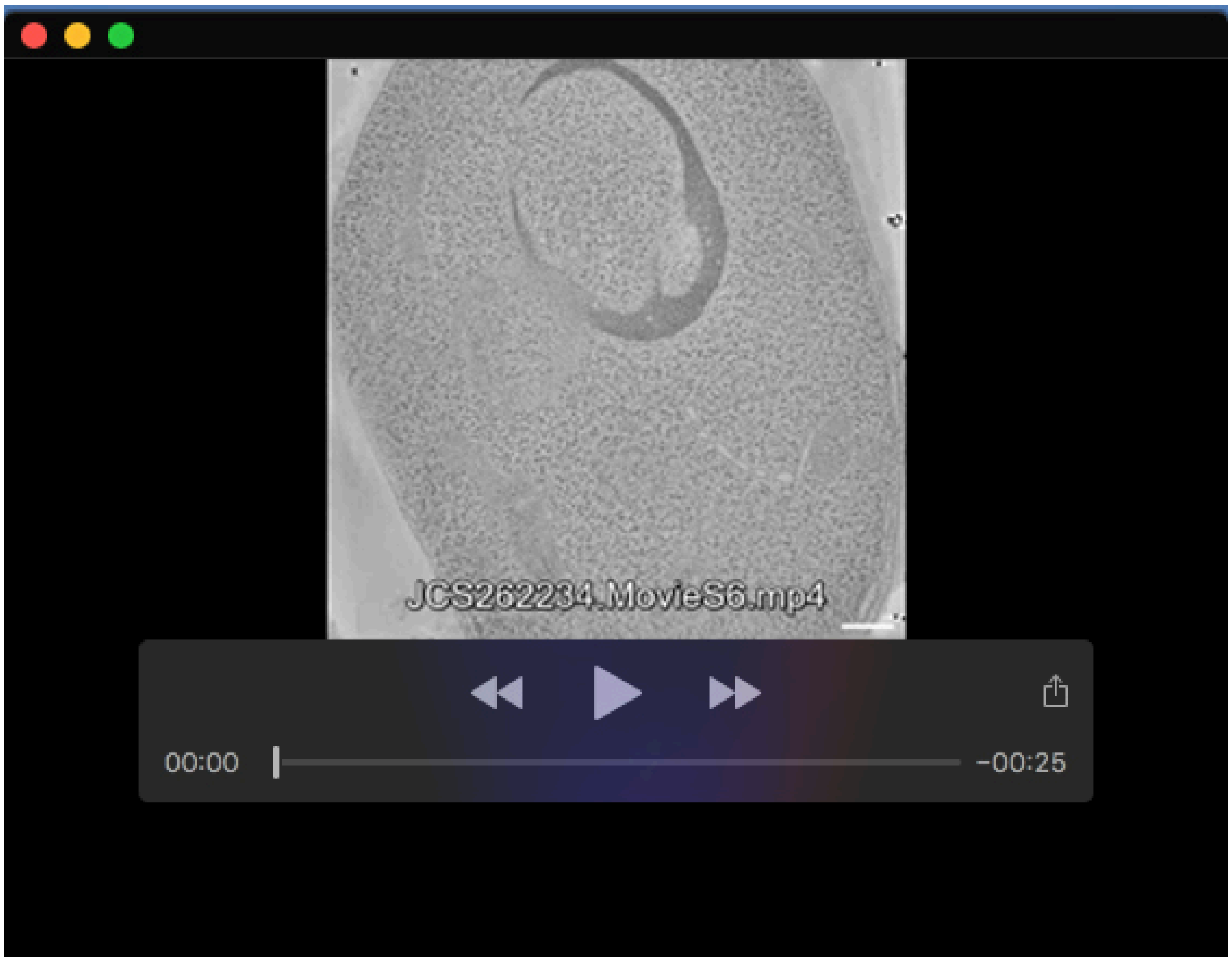

Movie 6. EM tomography data showing 3-D renderings of Golgi, vacuoles, and vesicles in *apl6*Δ cells. Flat Golgi shown in magenta, round Golgi shown in cyan, curved Golgi shown in purple, vacuole membrane shown in red, and cytosolic vesicles shown in grey. Scale bars show 200 nm when zoomed out (beginning) and 100 nm when zoomed in (end).

Journal of Cell Science • Supplementary information

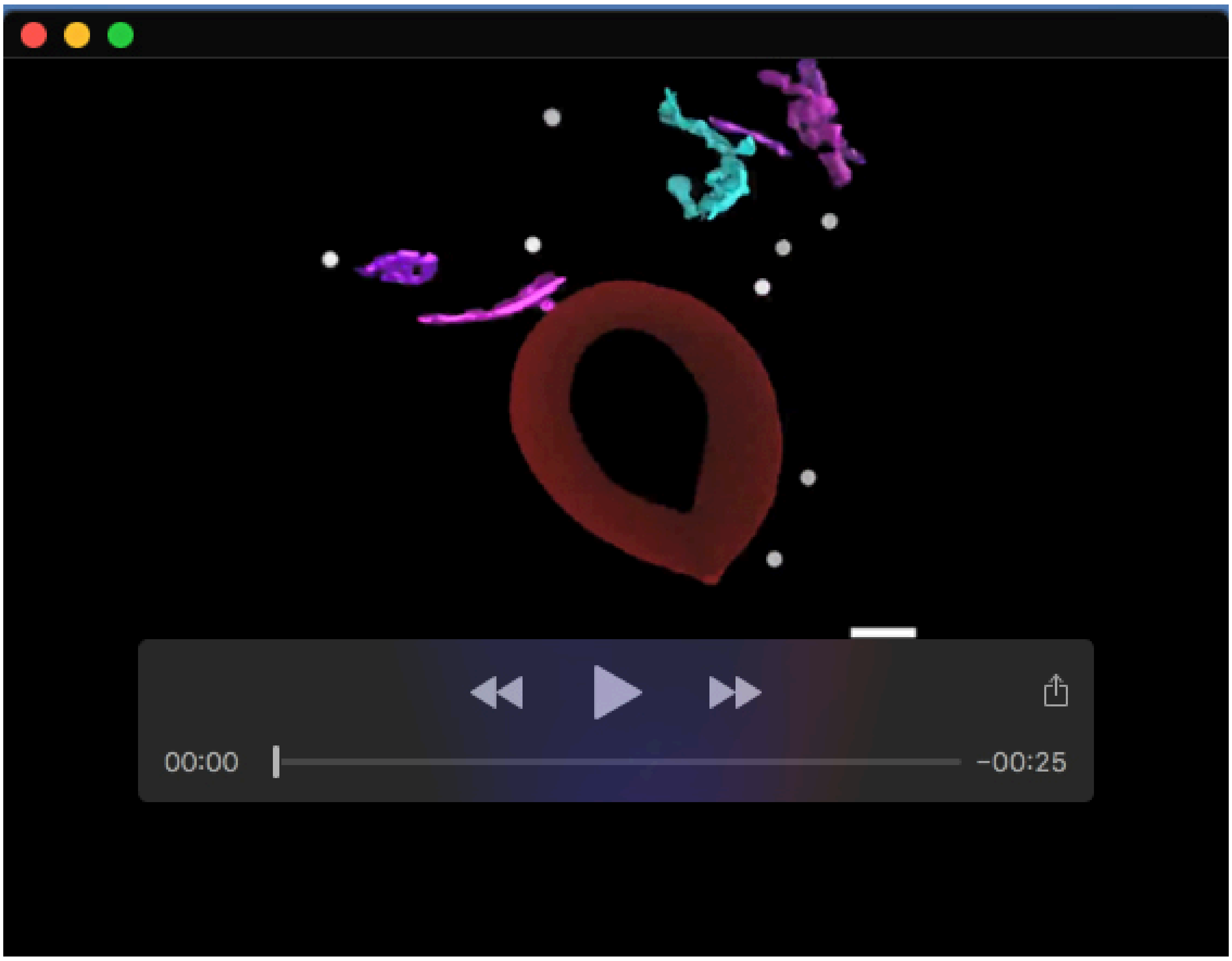

Movie 7. EM tomography data showing 3-D renderings of Golgi, vacuoles, and vesicles in *apl6-743*Δ cells. Flat Golgi shown in magenta, round Golgi shown in cyan, curved Golgi shown in purple, vacuole membrane shown in red, and cytosolic vesicles shown in grey. Scale bars show 200 nm when zoomed out (beginning) and 100 nm when zoomed in (end).

Journal of Cell Science • Supplementary information

Supplementary References

Angers, C. G. and Merz, A. J. (2009). HOPS Interacts with Apl5 at the Vacuole Membrane and Is Required for Consumption of AP-3 Transport Vesicles. *MBoC* 20, 4563–4574.

Costaguta, G., Stefan, C. J., Bensen, E. S., Emr, S. D. and Payne, G. S. (2001). Yeast Gga Coat Proteins Function with Clathrin in Golgi to Endosome Transport. *Molecular Biology of the Cell* **12**, 1885.

Costaguta, G., Duncan, M. C., Fernández, G. E., Huang, G. H. and Payne, G. S. (2006). Distinct Roles for TGN/Endosome Epsin-like Adaptors Ent3p and Ent5p. *MBoC* **17**, 3907–3920.

Cowles, C. R., Odorizzi, G., Payne, G. S. and Emr, S. D. (1997). The AP-3 Adaptor Complex Is Essential for Cargo-Selective Transport to the Yeast Vacuole. *Cell* **91**, 109–118.

Day, K. J., Casler, J. C. and Glick, B. S. (2018). Budding Yeast Has a Minimal Endomembrane System. *Dev Cell* 44, 56-72.e4.

Gustafson, M. A. and Fromme, J. C. (2017). Regulation of Arf activation occurs via distinct mechanisms at early and late Golgi compartments. *Mol Biol Cell* **28**, 3660–3671.

Highland, C. M. and Fromme, J. C. (2021). Arf1 directly recruits the Pik1-Frq1 PI4K complex to regulate the final stages of Golgi maturation. *MBoC* **32**, 1064–1080.

Johnson, L. M., Bankaitis, V. A. and Emr, S. D. (1987). Distinct sequence determinants direct intracellular sorting and modification of a yeast vacuolar protease. *Cell* **48**, 875–885.

Payne, G. S. and Schekman, R. (1989). Clathrin: a role in the intracellular retention of a Golgi membrane protein. *Science* **245**, 1358–1365.

Robinson, J. S., Klionsky, D. J., Banta, L. M. and Emr, S. D. (1988). Protein sorting in Saccharomyces cerevisiae: isolation of mutants defective in the delivery and processing of multiple vacuolar hydrolases. *Mol. Cell. Biol.* **8**, 4936.

Sheffield, P., Garrard, S. and Derewenda, Z. (1999). Overcoming Expression and Purification Problems of RhoGDI Using a Family of "Parallel" Expression Vectors. *Protein Expression and Purification* **15**, 34–39.

Tan, S. (2001). A Modular Polycistronic Expression System for Overexpressing Protein Complexes in Escherichia coli. *Protein Expression and Purification* **21**, 224–234.

Thomas, L. L. and Fromme, J. C. (2016). GTPase cross talk regulates TRAPPII activation of Rab11 homologues during vesicle biogenesis. *Journal of Cell Biology* **215**, 499–513.

Wang, N., Lo Presti, L., Zhu, Y.-H., Kang, M., Wu, Z., Martin, S. G. and Wu, J.-Q. (2014). The novel proteins Rng8 and Rng9 regulate the myosin-V Myo51 during fission yeast cytokinesis. *J Cell Biol* **205**, 357–375.

Journal of Cell Science • Supplementary information
