# Supplementary material for: Disordered hinge regions of the AP-3 adaptor complex promote vesicle budding from the late Golgi in yeast
Source: J Cell Sci. 2024 Nov 8;137(21):jcs262234. doi: 10.1242/jcs.262234 (PMC11574352; doi:10.1242/jcs.262234)
Supplement: Supplementary file 1 [file joces-137-262234_review_history.pdf]

Disordered hinge regions of the AP-3 adaptor complex promote vesicle budding from the late Golgi in yeast

Mitchell Leih, Rachael L. Plemel, Matt West, Cortney G. Angers, Alexey J. Merz and Greg Odorizzi
DOI: 10.1242/jcs.262234

Editor: Robert Parton

Review timeline
Original submission: 30 April 2024
Editorial decision: 11 June 2024
First revision received: 27 August 2024
Accepted: 21 September 2024

Original submission

First decision letter

MS ID#: JOCES/2024/262234

MS TITLE: Disordered hinge regions of the AP-3 adaptor complex are needed for vesicle budding from the late Golgi in yeast

AUTHORS: Mitchell Leih, Rachael Plemel, Matt West, Cortney Angers, Alexey J Merz, and Greg Odorizzi

We have now reached a decision on your manuscript. As you will see the reviewers all felt that your manuscript was potentially suitable for publication in the Journal of cell Science but they did raise a number of issues that would need to be addressed before I can recommend acceptance. In particular, the reported effects on Golgi morphology have been questioned and you might want to reconsider these conclusions or carry out further experiments. As you will see there are also questions about cargo being retained in the Golgi by AP3 complexes lacking the ear domains which may require further experimentation. I hope that you can address these concerns and the other issues raised by the referees and look forward to receiving a revised manuscript in due course. I will evaluate whether the manuscript needs to go back to all three reviewers at that time.

To see the reviewers' reports and a copy of this decision letter, please go to: https://submit-jcs.biologists.org and click on the 'Manuscripts with Decisions' queue in the Author Area. (Corresponding author only has access to reviews.)

Please ensure that you clearly highlight all changes made in the revised manuscript. Please avoid using 'Tracked changes' in Word files as these are lost in PDF conversion.

I should be grateful if you would also provide a point-by-point response detailing how you have dealt with the points raised by the reviewers in the 'Response to Reviewers' box. Please attend to all of the reviewers' comments. If you do not agree with any of their criticisms or suggestions please explain clearly why this is so.

Reviewer 1

© 2024. Published by The Company of Biologists under the terms of the Creative Commons Attribution License (https://creativecommons.org/licenses/by/4.0/).

Advance summary and potential significance to field

In this manuscript, Leih and colleagues present compelling data that the unstructured “hinge” regions of the yeast AP-3 adaptor complex are required for function of the AP-3 complex. Using multi-color fluorescent imaging to look at AP-3 colocalization in cells, the authors show that AP-3 is efficiently recruited to membranes, but is aberrantly accumulated in the Golgi, presumably because AP-3 coated vesicles cannot bud efficiently without hinges. Using chromogenic assays they show that only transport of AP-3 cargo is affected, while secretion through the CPY pathway is unaffected. More broadly, this study nicely sets the stage for further investigation of the mechanisms for these regions of the complex. As AP-3 is an “ancient” vesicle coat, understanding these mechanisms will be of broad interest to those broadly studying vesicular transport. Overall, this study provides a new insight that is well supported and technically sound. I have only minor comments and believe this manuscript is well suited for publication.

Comments for the author

Minor comments:

- In Figure 1A, I think it would be very helpful to “stretch” out the structures in Chimera or another program to better show the structural differences between the large adaptins. The placement of domains that do not interact is essentially random in AF structures, and it tends to make a fuzzy ball of disordered regions. This could be done manually in Chimera, but can also be done automatically using this software: https://github.com/ryanemenecker/dodo. Barring that, color coding the trunk, hinge, and ears could help with legibility.

- In Supp. Fig. 2, I notice that the Apl5 truncations do not follow a clean pattern where short truncation = small phenotype and long truncation = large phenotype, whereas in Apl6 this seems to be the case. For Apl5, the 856delta is intermediate compared to the 918delta, which is a short truncation but has a significant phenotype. Is there an explanation for this? Does this correlate with binding to Vps41 (the 1999 Rehling/Emr paper shows that a short C-terminal region mediates binding to Vps41 by Y2H). Looking at the Uniprot AF model for Apl5 these two constructs seem to be immediately before and immediately after a long helix. Is there an explanation for this?

- In Supp. Fig 2, an Apl5 construct is listed as 713delta, but 710 is in text and main figures. Which is correct? Or where two different constructs used?

- In Figure 4, I thought the coloring was hard to immediately understand and the green data bars look black. A new color or simply changing the line thickness might make the difference easier to read.

- In the discussion, I’m not sure if this claim is rightly supported ->

Curiously, while we found yeast AP-3 function was disabled by truncating either of its large subunits, the function of AP-2 in HeLa cells was found to be unaffected by truncation of *α*2 or by mutations in accessory- binding sites that had been identified in this subunit (Motley et al., 2006), suggesting that mammalian AP-2 large subunits harbor a level of functional redundancy not present in yeast AP-3.
This finding was followed up in two subsequent papers, (https://www.sciencedirect.com/science/article/pii/S1534580713003821 and https://www.embopress.org/doi/full/10.15252/embj.201591518). They show that while deleting the alpha appendage doesn’t affect Transferrin uptake, it essentially makes an unregulated AP2 that has very altered CCP dynamics. So while Tfn uptake is the same, regulated endocytosis is altered and signaling through CME-regulated receptors is affected.

Altering the language to say something more akin to “deleting the alpha appendage alters AP-2 regulation, but otherwise does not affect the ability to successfully transport cargo” would be more precise.

<u>Reviewer 2</u>

© 2024. Published by The Company of Biologists under the terms of the Creative Commons Attribution License (https://creativecommons.org/licenses/by/4.0/).

Advance summary and potential significance to field

The manuscript of Leih et al focus on the role of the unstructured ear domains of the large AP-3 subunits Apl5 and Apl6. Unlike other AP complexes, yeast AP-3 has apparently no folded ear domains at their C-terminal. This then poses the question, whether such domains are even required for AP-3 function. Here the authors show that Apl5 and Apl6 mutants lacking the C-terminal parts still seem to form an AP-3 complex, even though this complex is not budding from the Golgi. Instead, AP-3 dependent budding is inhibited and AP-3 cargoes (here a fusion protein of Nyv1 and Snc1) is instead sorted to the plasma membrane. Colocalization experiments confirm signals of AP-3 subunits at the Golgi, which seems to change in morphology due to the lack of AP-3 mediated transport. Interestingly, budding from the TGN via the Gga complex is not impaired, indicating a specific defect of AP-3 specific transport, which is nicely demonstrated also by the high-resolution mobility analysis of Apl5-GFP. The data overall agree with a model, where the hinge domains are essential for the formation of AP-3 vesicles at the Golgi.

This is an overall very nice and almost complete study, where the authors take their available repertoire of techniques to dissect the consequences of the Apl5 and Apl6 truncations. Their data clearly show that AP-3 with truncated subunits still associates with the Golgi, but is unable to function in budding. What is missing in my view is the question of assembly of the AP-3 complex (I assume it is assembled) and the possible role of the missing domains, which the authors can only speculate on. It would also be interesting to know if the arrested AP-3 is present in complexes with cargo that is now trapped at the Golgi.

Comments for the author

Specific questions:

1. The authors use CMAC stained vacuoles as an orientation when they show tagged subunits of AP-3 and other proteins. I would appreciate it if they would also provide in each fluorescent image an outline of the cell like they do in Figure 4.
2. Multiple lines of evidence show that the AP-3 complexes arrest at the Golgi, when the ear domains are missing. I am wondering if this traps cargo. One experiment would be to purify Apl5 via GFP beads from either truncated or full-length proteins and then analyze the eluate by mass spectrometry. This would address two questions of this study: First, is the complex still intact (I assume so) and second, what is arrested in the truncated complex under these conditions at the Golgi. The authors also could use the eluate to test for possible cargo associations by Western blot.
3. Figure 5D,E: I recommend labeling the structures and provide a legend next to the figure. It makes the figure more accessible. How do the authors know that this is Golgi that they are assigning here in color? I did not see any correlative microscopy.

<u>Reviewer 3</u>

Advance summary and potential significance to field

In this manuscript, Leih et al. investigate the function of the hinge regions of yeast AP-3 large subunits. They first show by computational modelling that these regions, which are disordered, to not connect to ear domains typical for AP complexes. They also show that these hinges do not bind clathrin.
Then, they dissect how these hinges influence localisation and dynamics of AP3-dependent vesicle trafficking at the Golgi. They conclude that either of the two hinges is required for AP3-dependent budding, as in hinge-deficient cells, AP-3 accumulates in Golgi compartments and shows decreased mobility.
This paper provides novel mechanistic insights into the function of AP-3. It is a very interesting study, the data is mostly very convincing and well presented, the paper is well written, and the results are an important contribution to the membrane trafficking field. I have a few Comments for the authors to consider, and overall recommend the paper to be published in JCS upon revisions.

© 2024. Published by The Company of Biologists under the terms of the Creative Commons Attribution License (https://creativecommons.org/licenses/by/4.0/).

Comments for the author

Major points:
I find most of the results very convincing, except for the results leading to the conclusion that Golgi morphology is affected when hinge regions are missing (Figure 5). The basis of this conclusion is two-fold: the fluorescence images in Fig 5A which supposedly show differences in morphology of the structures and the electron tomography data in Fig 5D and E. I don't find these differences in the fluorescence images obvious, and the quantification of the EM data is not statistically significant. Thus, I find that the basis for this conclusion is very weak. I would recommend the authors to reconsider this conclusion.

Discussion: The redundancy of the two hinges is not addressed by the experiments, nor is it considered in the Discussions. Do the authors have any hypothesis why the two hinges appear to be entirely redundant in their function? Or are there potential subtle differences to be discussed?
Page 13, third and second last lines: "…. that the unstructured hinges of AP-3 have a direct role in membrane remodeling and fission…." Another (not mutually exclusive) possibility to consider is that the unstructured regions help dispersing AP-3 on the membrane and thereby promote dynamics, in some analogy to what was shown for OSBP at membrane contact sites (Jamecna et al. Dev Cell 2019).

Minor points:
Figure 2C: I would recommend showing the single-channel images in grayscale rather than in green and blue; it would improve visibility.
Similarly, in other Figures where single channels (green and magenta) are shown together with the blue channel and then there is an additional merge image, I would recommend showing the channel of interest in grayscale, even when combined with blue for CMAC. For example, Fig 3A left panel: the Apl5-GFP signal in grayscale, CMAC in blue; middle panel: Sec7-MARS in grayscale, CMAC in blue; right panel: in 3 colours as now.
Figure 5D and E: The tomography results would be better presented if in addition, higher magnification images (zoom-ins) would be shown of the structures of interest.

Superresolution (SoRa): The abbreviation SoRa is a bit cryptic, what does it stand for?

Suppl. Fig 4 legend to I and J: "number of AP-3 puncta" you mean gold beads, not puncta, right? Puncta is otherwise used for fluorescent signals throughout the manuscript.

Page 8, first paragraph, in the sentence starting with "Clusters of AP-3 with Sec7 were seen in >90% of cells… can you indicate n number of cells and experiments?

Page 9, first paragraph: the dynamics are nicely presented in the movies 1 and 2, but the manuscript would benefit if additionally, series of stills (e.g with arrows pointing at puncta changing position or immobile puncta) could be shown in Figure 4.

First revision

<u>Author response to reviewers' comments</u>

We thank the reviewers and the monitoring editor for careful examination of our manuscript. Listed below, each reviewer comment (*italicized and bounded by quotation marks*) is followed by our response (bulleted (•) and written in plain text). The page number where an edit is located in the revised manuscript is identified in each response, and each of these edits is highlighted the revised manuscript. Please note we made a modest change in the title:

© 2024. Published by The Company of Biologists under the terms of the Creative Commons Attribution License (https://creativecommons.org/licenses/by/4.0/).

Original title: “Disordered hinge regions of the AP-3 adaptor complex are needed for vesicle budding from the late Golgi in yeast”

Revised title: “Disordered hinge regions of the AP-3 adaptor complex promote vesicle budding from the late Golgi in yeast”

REVIEWER 1

“In Figure 1A, I think it would be very helpful to “stretch” out the structures in Chimera or another program to better show the structural differences between the large adaptins…. Barring that, color coding the trunk, hinge, and ears could help with legibility.”

- As suggested, the colors for the ribbon structures in Figure 1 and Supplemental Figure 1 were changed to distinguish the trunk, hinge, and ear domains of the large subunits of yeast AP complexes.

“In Supp. Fig. 2, I notice that the Apl5 truncations do not follow a clean pattern where short truncation = small phenotype and long truncation = large phenotype, whereas in Apl6 this seems to be the case. For Apl5, the 856delta is intermediate compared to the 918delta, which is a short truncation but has a significant phenotype. Is there an explanation for this? Does this correlate with binding to Vps41 (the 1999 Rehling/Emr paper shows that a short C-terminal region mediates binding to Vps41 by Y2H). Looking at the Uniprot AF model for Apl5, these two constructs seem to be immediately before and immediately after a long helix. Is there an explanation for this?”

- It is unclear why the cargo-sorting defect is worse in the two longer alleles than it is in *apl5-856Δ*, but the phenotype is reproducible. Because it was not central to the main point of this study, we chose not to comment on it in the original manuscript. However, in the revision, we point this out this discontinuity in the legend to Supplemental Figure 2B and conjecture that it, “might signify a structural distortion that impacts Apl5 function when intermediate lengths of the hinge region are present, though such a possibility would require further analysis.”

“In Supp. Fig 2, an Apl5 construct is listed as 713delta, but 710 is in text and main figures. Which is correct? Or where two different constructs used?”

- Yes, two different constructs were used, which we should have clarified in the original submission. While the survey of *apl5* truncation-mutant alleles by qualitative overlay included the *apl5-713Δ* allele, the experiments shown in the main set of figures used the *apl5-710Δ* allele, which was used for all other experiments in this study on the basis of our prior publication showing that the region of the hinge deleted in this allele corresponds to a fragment of Apl5 (amino acids 711-932) that directly bind the Vps41 subunit of the HOPS complex that tethers AP-3 vesicles to vacuoles (Angers and Merz, 2009). This clarification is now stated in the legend to Supplemental Figure 2, where we also note that the amount of GNSS missorted by *apl5-713Δ* cells, which was qualitatively detected by overlay (Supplemental Figure 2B), is consistent with our finding by quantitative assay that 80% of GNSS is missorted by *apl5-710Δ* cells (Figure 2B).

“In Figure 4, I thought the coloring was hard to immediately understand, and the green data bars look black. A new color or simply changing the line thickness might make the difference easier to read.”

- The dark green data bars were switched for a lighter shade to provide contrast for the graphs shown in Figure 4C

“In the discussion, I’m not sure if this claim is rightly supported ->

‘Curiously, while we found yeast AP-3 function was disabled by truncating either of its large subunits, the function of AP-2 in HeLa cells was found to be unaffected by truncation of a2 or by mutations in accessory- binding sites that had been identified in this subunit (Motley et al., 2006), suggesting that mammalian AP-2 large subunits harbor a level of functional redundancy not present in yeast AP-3.’

© 2024. Published by The Company of Biologists under the terms of the Creative Commons Attribution License (https://creativecommons.org/licenses/by/4.0/).

This finding was followed up in two subsequent papers….They show that while deleting the alpha appendage doesn't affect Transferrin uptake, it essentially makes an unregulated AP2 that has very altered CCP dynamics. So while Tfn uptake is the same, regulated endocytosis is altered and signaling through CME-regulated receptors is affected. Altering the language to say something more akin to "deleting the alpha appendage alters AP-2 regulation, but otherwise does not affect the ability to successfully transport cargo" would be more precise."

- We agree with the recommendation and have edited accordingly (p. 13): "Curiously, cargo endocytosis in HeLa cells was unaffected by truncation of the AP-2 α2 subunit or by mutations in accessory-binding sites that had been identified in this subunit (Motley et al., 2006), though α2 truncation altered the kinetics of endocytic vesicle formation and disrupted events downstream of vesicle budding (Aguet et al., 2013; Reis et al., 2015)."

REVIEWER 2

"What is missing in my view is the question of assembly of the AP-3 complex (I assume it is assembled) and the possible role of the missing domains, which the authors can only speculate on."

- Evidence that neither the Apl5 nor Apl6 truncation disables AP-3 complex assembly includes 1) the localization of Apl5- and Apl6-GFP to Golgi puncta in truncation-mutant cells but not in deletion-mutant cells lacking any one subunit of the heterotetramer (stated more explicitly in the revised manuscript on pp. 7-8); 2) our unpublished mass-spec analysis of immuno-isolated AP-3 complexes in yeast expressing wild-type or truncated subunits (now stated in the revised manuscript on p. 13); and 3) the absence of hinge sequences in the published cryo-EM structure of wild-type yeast AP-3 complexes (Schoppe et al., 2021), which is consistent with the hinges being disordered and not contributing to quaternary structure (now stated in the revised manuscript on p. 5). We do suggest speculative models for the functions of the hinge regions in paragraphs 3-5 of the discussion in the original and revised manuscript.

"It would also be interesting to know if the arrested AP-3 is present in complexes with cargo that is now trapped at the Golgi."

- We did not detect enrichment of known AP-3 cargo proteins (e.g., Pho8 [aka ALP] or Sna4) in our unpublished mass-spec analysis of immuno-isolated AP-3 complexes in yeast expressing truncated AP-3 relative to complexes isolated from yeast expressing wild-type AP-3 subunits. Likewise, we have observed no enrichment of the Pho8 cargo protein in our unpublished anti-Pho8 western blots of anti-GFP pulldowns from yeast lysates expressing Apl5-GFP (or Apl6-GFP) in wild-type or truncated Apl6 (or Apl5). Moreover, we did not observe the GNSS cargo protein at intracellular puncta in AP-3 truncation-mutant cells (Figure 2), as would have been expected if truncated/unbudded AP-3 complexes trap cargoes. This topic is now addressed in the revised manuscript on p. 13: "We found no evidence that unbudded AP-3 complexes trap cargoes, based on our observations that the truncation mutations do not cause the GNSS cargo to accumulate at the Golgi (Figure 2) or cause enhanced recovery of cargoes with immuno-isolated AP-3 complexes analyzed by mass spectrometry and by western blot (unpublished results)." We also did not observe Pho8 accumulation in the 100,000 x *g* pellet that contains Golgi membranes following differential centrifugation of yeast lysates (unpublished), though this is a less direct way of addressing the possibility that AP-3 hinge truncations trap cargoes at the Golgi.

"The authors use CMAC stained vacuoles as an orientation when they show tagged subunits of AP-3 and other proteins. I would appreciate it if they would also provide in each fluorescent image an outline of the cell like they do in Figure 4."

- As requested, these cell outlines are now provided in each fluorescence image except for a selection in Figure 2C, where the GNSS reporter shows the cell outline upon cargo missorting.

"Multiple lines of evidence show that the AP-3 complexes arrest at the Golgi, when the ear domains are missing. I am wondering if this traps cargo. One experiment would be to purify Apl5 via GFP beads from either truncated or full-length proteins and then analyze the eluate by mass spectrometry. This would address two questions of this study: First, is the complex still intact (I

© 2024. Published by The Company of Biologists under the terms of the Creative Commons Attribution License (https://creativecommons.org/licenses/by/4.0/).

assume so) and second, what is arrested in the truncated complex under these conditions at the Golgi. The authors also could use the eluate to test for possible cargo associations by Western blot."

- The same suggestions were (inadvertently, we assume) made by the same reviewer above. Our responses are provided in the initial queries there.

"Figure 5D,E: I recommend labeling the structures and provide a legend next to the figure. It makes the figure more accessible. How do the authors know that this is Golgi that they are assigning here in color? I did not see any correlative microscopy."

- As recommended, we have added labeling and a graphical legend to Figure 5. Regarding our confidence in structure identity, our unpublished work on understanding organelle morphologies in yeast has included using immuno-EM to identify Golgi membrane structures in serial thick sections that we then analyze by tomography. The rounded vs flat Golgi compartments reported herein match all of our previously observed parameters for Golgi compartments and, moreover, in yeast we have observed no organelle morphologies like those of the Golgi. All other organelles can be identified by their high-resolution morphologies and similarly be interpreted, identified, and excluded. Further, immuno-label can be patchy on Golgi in thin sections prepared for EM, so no definitive label could be used with our method of sectioned samples, and the small size of the round Golgi in our data set proves difficult to image and analyze unless using a multi-step method like ours.

REVIEWER 3

"I find most of the results very convincing, except for the results leading to the conclusion that Golgi morphology is affected when hinge regions are missing (Figure 5). The basis of this conclusion is two-fold: the fluorescence images in Fig 5A which supposedly show differences in morphology of the structures and the electron tomography data in Fig 5D and E. I don't find these differences in the fluorescence images obvious, and the quantification of the EM data is not statistically significant. Thus, I find that the basis for this conclusion is very weak. I would recommend the authors to reconsider this conclusion."

- On p. 10 of the revised manuscript, we more explicitly state the change in Gea2 and Sec7 distribution patterns in response to AP-3 truncation: "When either hinge of AP-3 is truncated, spatial separation between Gea2-mNeon and Sec7-MARS is maintained (Fig. 5B,C), but each exhibits radial patterning. This patterning was unlike the punctate or tubular shapes observed in wild-type cells, but similar to what was observed for each GEF when coexpressed with GFP-tagged AP-3 in *apl6* or *apl5* truncation-mutant cells (Fig. 3)." As suggested, we have softened the interpretation of how AP-3 truncation affects Golgi morphology. In addition to stating that the membrane surface-area changes measured by tomography were not statistically significant, we state on p. 10 of the revised manuscript that the higher frequency of immunogold labeling for AP-3 on rounded compartments suggests, "unbudded AP-3 complexes might prefer curved Golgi membranes, though more direct experimentation would be needed to determine the extent to which AP-3 has this property."

"Discussion: The redundancy of the two hinges is not addressed by the experiments, nor is it considered in the Discussions. Do the authors have any hypothesis why the two hinges appear to be entirely redundant in their function? Or are there potential subtle differences to be discussed?"

In the revised manuscript (pp. 14-15), we have included a paragraph in the discussion proposing that the hinges of Apl5 and Apl6 are not redundant, even though each is required for AP-3 vesicle budding: "The Vps41 binding site in AP-3 is located in the hinge sequence deleted in *apl5-710Δ*. Though our results clearly show Apl5 truncation inhibits AP-3 vesicle budding from the Golgi, defective AP-3 cargo sorting also occurs upon genetic disruption of membrane fusion at the vacuole (Schwartz et al., 2017; Plemel et al., 2021). This fusion step relies on HOPS-mediated tethering of AP-3 vesicles to the vacuole membrane (Angers and Merz, 2009). Thus, the defect in AP-3 vesicle budding combined with the loss of Vps41 binding to AP-3 in *apl5-710Δ* cells could explain why this mutant strain has a stronger cargo-missorting phenotype (~80% missorted GNSS) compared to *apl6-743Δ* cells (~50% missorted GNSS), though testing this hypothesis would require creative restoration of the Vps41 binding site in cells expressing the truncated Apl5 protein. In either case, the different

© 2024. Published by The Company of Biologists under the terms of the Creative Commons Attribution License (https://creativecommons.org/licenses/by/4.0/).

severity to which truncation of Apl5 vs Apl6 affects AP-3 cargo sorting suggests that the hinge regions are not functionally redundant, even though truncation of either hinge impairs the budding of AP-3 vesicles. We anticipate that the Apl5 and Apl6 hinges bind different sets of accessory proteins involved in AP-3 vesicle trafficking, though some interaction partners might be shared, for example as has been observed for hinge-ear regions of other AP complexes (Traub, 2005).”

“Page 13, third and second last lines: “…. that the unstructured hinges of AP-3 have a direct role in membrane remodeling and fission….” Another (not mutually exclusive) possibility to consider is that the unstructured regions help dispersing AP-3 on the membrane and thereby promote dynamics, in some analogy to what was shown for OSBP at membrane contact sites (Jamecna et al. Dev Cell 2019).”

- That is a point we should have considered. In the revised manuscript, this possibility is discussed (p. 14): “Another possibility is that the biophysical properties of the disordered hinges control the mobility and/or orientation of AP-3 at the membrane surface, as has been observed for the disordered N terminus of oxysterol-binding protein at membrane contact sites between the endoplasmic reticulum and the TGN (Jamecna et al., 2019).”

“Figure 2C: I would recommend showing the single-channel images in grayscale rather than in green and blue; it would improve visibility. Similarly, in other Figures where single channels (green and magenta) are shown together with the blue channel and then there is an additional merge image, I would recommend showing the channel of interest in grayscale, even when combined with blue for CMAC. For example, Fig 3A left panel: the Apl5-GFP signal in grayscale, CMAC in blue; middle panel: Sec7-MARS in grayscale, CMAC in blue; right panel: in 3 colours as now.”

- We hope it is permissible for us not to follow this recommendation, as we believe the color images adequately display the protein localizations in these images.

“Figure 5D and E: The tomography results would be better presented if in addition, higher magnification images (zoom-ins) would be shown of the structures of interest.”

- Adding higher magnification images would make this figure massive. Instead, we now include in the supplement movie files of the tomography of the *APL6*, *apl6-743Δ* and *apl6Δ* strains as representatives for AP-3 subunit mutations because these movies show zoomed-in and rotated Golgi structures in each strain.

“Superresolution (SoRa): The abbreviation SoRa is a bit cryptic, what does it stand for?”

- SoRa (super-resolution optical reassignment) is the method for super-resolution used by the Yokogawa spinning disk technology. To eliminate confusion, we now refer to this term only once in the Materials and Methods of the revised manuscript (p. 19). Elsewhere, we simply state, “super-resolution confocal fluorescence microscopy.” Because these instances are numerous, their locations are not marked in the margin of the revised manuscript.

“Suppl. Fig 4 legend to I and J: “number of AP-3 puncta” you mean gold beads, not puncta, right? Puncta is otherwise used for fluorescent signals throughout the manuscript.”

- Correct – thanks for catching this mistake. That supplemental figure legend has been reworded accordingly: “Quantitation of the number of gold particles observed by immuno-EM using anti-GFP antibodies.”

“Page 8, first paragraph, in the sentence starting with “Clusters of AP-3 with Sec7 were seen in >90% of cells… can you indicate n number of cells and experiments?”

- For each experiment using fluorescence microscopy, at least 200 cells were sampled. This is now indicated on p. 8. “Clusters of AP-3 with Sec7 were seen in >90% of *apl6-743Δ* and *apl5-710Δ* mutant cells, but such clusters were observed at a low frequency (~5%) in wild-type cells expressing full-length AP-3 subunits (Figure 3C; n>200).”

“Page 9, first paragraph: the dynamics are nicely presented in the movies 1 and 2, but the manuscript would benefit if additionally, series of stills (e.g with arrows pointing at puncta changing position or immobile puncta) could be shown in Figure 4.”

© 2024. Published by The Company of Biologists under the terms of the Creative Commons Attribution License (https://creativecommons.org/licenses/by/4.0/).

- Although we agree with the logic of adding stills to show puncta dynamics, several problems arise when we consider this option. 1) Because of the speed at which each frame was taken, a single second of Apl5-GFP puncta dynamic movement would represent about 20 stills, making the figure dimensions much larger than they currently are. 2) The lifespan of the puncta in these movies varies greatly from transient (a few frames) to very long-lived (the full 30 seconds). We feel that choosing individual puncta to be represented in the stills would be disingenuous to the data. The movies provided in the supplement, paired with the particle tracking and displacement maps shown in the main figure, give the reader a more complete representation of the puncta dynamics.

Second decision letter

MS ID#: JOCES/2024/262234

MS TITLE: Disordered hinge regions of the AP-3 adaptor complex promote vesicle budding from the late Golgi in yeast

AUTHORS: Mitchell Leih, Rachael Plemel, Matt West, Cortney Angers, Alexey J Merz, and Greg Odorizzi

I am happy to tell you that your manuscript has been accepted for publication in Journal of Cell Science, pending standard publication integrity checks.

Reviewer 1

Advance summary and potential significance to field

See previous review:

In this manuscript, Leih and colleagues present compelling data that the unstructured “hinge” regions of the yeast AP-3 adaptor complex are required for function of the AP-3 complex. Using multi-color fluorescent imaging to look at AP-3 colocalization in cells, the authors show that AP-3 is efficiently recruited to membranes, but is aberrantly accumulated in the Golgi, presumably because AP-3 coated vesicles cannot bud efficiently without hinges. Using chromogenic assays they show that only transport of AP-3 cargo is affected, while secretion through the CPY pathway is unaffected. More broadly, this study nicely sets the stage for further investigation of the mechanisms for these regions of the complex. As AP-3 is an “ancient” vesicle coat, understanding these mechanisms will be of broad interest to those broadly studying vesicular transport. Overall, this study provides a new insight that is well supported and technically sound.

Comments for the author

The authors have addressed all of my comments and concerns. Their response to the other reviewers seems adequate. This paper is ready for publication.

-Rick Baker
UNC Chapel Hill

Reviewer 2

Advance summary and potential significance to field

The authors addressed all my concerns and have answered all questions regarding AP-3 assembly and possible cargo interaction. I therefore have no further requests and recommend publication of this complete work.

© 2024. Published by The Company of Biologists under the terms of the Creative Commons Attribution License (https://creativecommons.org/licenses/by/4.0/).

Comments for the author

I have no further requests.

Reviewer 3

Advance summary and potential significance to field

(Copied from my first review:)
In this manuscript, Leih et al. investigate the function of the hinge regions of yeast AP-3 large subunits. They first show by computational modelling that these regions, which are disordered, to not connect to ear domains typical for AP complexes. They also show that these hinges do not bind clathrin.
Then, they dissect how these hinges influence localisation and dynamics of AP3-dependent vesicle trafficking at the Golgi. They conclude that either of the two hinges is required for AP3-dependent budding, as in hinge-deficient cells, AP-3 accumulates in Golgi compartments and shows decreased mobility.
This paper provides novel mechanistic insights into the function of AP-3. It is a very interesting study, the data is mostly very convincing and well presented, the paper is well written, and the results are an important contribution to the membrane trafficking field.

Comments for the author

In this revised version of the manuscript and the rebuttal, the authors have adequately addressed all my comments. In my view the manuscript can now be published in JCS. I look forward to seeing it published and congratulate the authors on this very interesting work!

© 2024. Published by The Company of Biologists under the terms of the Creative Commons Attribution License (https://creativecommons.org/licenses/by/4.0/).
